# Splicing factor SRSF1 attenuates cardiomyocytes apoptosis via regulating alternative splicing of *Bcl2L12*

**DOI:** 10.1186/s13578-024-01324-3

**Published:** 2024-11-22

**Authors:** Yilin Xie, Zhenbo Yang, Wenxian Chen, Changsheng Zhong, Mengyang Li, Lei Zhang, Ting Cheng, Qin Deng, Huifang Wang, Jin Ju, Zhimin Du, Haihai Liang

**Affiliations:** 1 Zhuhai People’s Hospital (Zhuhai Clinical Medical College of Jinan University), Guangdong Provincial Key Laboratory of Tumor Interventional Diagnosis and Treatment, Jinan University, Zhuhai, 519000 Guangdong China; 2 State Key Laboratory of Quality Research in Chinese Medicines, Macau University of Science and Technology, Macau , 999078 China; 3 Department of Pharmacy, The Third Affiliated Hospital (The Affiliated Luohu Hospital) of Shenzhen University, Shenzhen, Guangdong, 518000 China; 4 State Key Laboratory of Frigid Zone Cardiovascular Diseases (SKLFZCD), Department of Pharmacology (State Key Labratoray -Province Key Laboratories of Biomedicine Pharmaceutics of China, Key Laboratory of Cardiovascular Research, Ministry of Education), College of Pharmacy, Harbin Medical University, Harbin, 150081 China; 5 College of Pharmacy, Shenzhen University Medical School, Shenzhen University, Shenzhen, 518055 Guangdong China; 6 College of Basic Medical Sciences, Shenzhen University Medical School, Shenzhen University, Shenzhen, 518055 Guangdong China; 7 Research Unit of Noninfectious Chronic Diseases in Frigid Zone (2019RU070), Chinese Academy of Medical Sciences, Harbin, 150081 China

**Keywords:** Alternative splicing, SRSF1, Myocardial infarction, Apoptosis, Bcl2L12

## Abstract

**Background:**

Aberrant alternative splicing (AS) events, triggered by the alterations in serine/arginine splicing factor 1 (SRSF1), a member of the SR protein family, have been implicated in various pathological processes. However, the function and mechanism of SRSF1 in cardiovascular diseases remain unclear.

**Results:**

In this study, we found that the expression of SRSF1 was significantly down-regulated in the hearts of mice with acute myocardial infarction (AMI) and H9C2 cells exposed to H_2_O_2_. Moreover, in vivo experiments utilizing adeno-associated virus serotype 9-mediated SRSF1 overexpression improved cardiac function and reduced infarct size in AMI mice. Mechanistically, we employed RNA-seq assay to identify AS aberrations associated with altered SRSF1 level in cardiomyocytes, and found that SRSF1 regulates the splice switching of Bcl2L12. Further study showed that silencing SRSF1 inhibits the inclusion of exon7 in Bcl2L12. Importantly, the truncated Bcl2L12 lacked the necessary structural elements and failed to interact with p53, thus compromising its ability to suppress apoptosis.

**Conclusions:**

Our study unraveled the role of SRSF1 as a splicing factor involved in the regulation of Bcl2L12 splice switching, thereby exerting an anti-apoptotic effect through the p53 pathway, which provides new insights into potential approaches targeting cardiomyocyte apoptosis in cardiovascular diseases.

**Supplementary Information:**

The online version contains supplementary material available at 10.1186/s13578-024-01324-3.

## Introduction

Acute myocardial infarction (AMI) is a major global health concern associated with high mortality and morbidity. Despite significant progress has been made in the early treatment of AMI, it still increases the risk of heart failure (HF) in patients, leading to higher long-term mortality rate [1, 2]. Therefore, it is imperative to further elucidating the pathogenesis of AMI and developing effective clinical interventions. Cardiomyocyte apoptosis plays a pivotal role in the pathogenesis of myocardial infarction and is triggered by factors such as oxidative stress, ischemia-reperfusion and hypoxia injury. This apoptosis leads to cardiomyocytes loss, cardiac remodeling, and ultimately aggravating cardiac insufficiency and heart failure [3, 4]. Hence, in-depth exploration of the mechanism of myocardial cell apoptosis in AMI to inhibit myocardial injury is crucial for improving the prognosis of AMI patients.

As one of the important mechanisms for gene regulatory variation in morphological evolution, alternative splicing (AS) has garnered considerable attention and research. AS is a process of post-transcriptional processing of pre-mRNA through seven distinct alternative splicing methods: skipping exon (SE), alternative 5′-splice site (A5SS), alternative 3′-splice site (A3SS), mutually exclusive exons (MEX), intron retention (IR), alternative 3′ untranslated regions (UTRs) and alternative first or last exon (AFE/ALE). These processes expand the coding ability of genes and enable a single gene to produce multiple transcripts and proteins [5, 6]. AS regulates approximately 90-95% of human genes, serving as a vital mechanism during development and facilitating responses to environmental changes at all stages of life [7, 8]. Similar to the importance of AS in the biological and physiological sense, the abnormalities of AS are often associated with various pathological processes. AS dysregulation is closely linked to nervous system diseases [9], aging [10], autoimmune diseases [11] and other processes. In addition, it has been shown that aberrant splicing mediated by mutations in cis-acting elements and dysregulation of splicing factors is prevalent in various cancer types [12].

The SR protein family, characterized by an RNA recognition motif (RRM) and one or more intrinsic disordered regions (IDRs) rich in alternating arginine and serine residues, are recognized to activate splicing by binding to exon enhancer sequences and recruitment of spliceosome [13, 14]. Among the SR protein members, SRSF1 (also known as ASF/SF2) was the first identified and has been implicated in the development and progression of glioma [15], breast cancer [5], lung cancer [16] and other tumor types by regulating gene splicing. In the context of cardiovascular diseases, studies have revealed that SRSF1 knockout mice exhibit aberrant splicing of Ca^2+^/calmodulin-dependent kinase IIδ in cardiomyocytes, leading to severe defects in myocardial excitation-contraction coupling [17]. Furthermore, SRSF1 can promote the proliferation of vascular smooth muscle through D133p53/KLF5 pathway [18]. Despite these findings, the role of SRSF1 in cardiovascular diseases, especially myocardial infarction, remains limited.

In the present study, we aimed to investigate the involvement of SRSF1 in myocardial infarction and unveil its anti-apoptotic role in cardiomyocytes. Our results revealed that SRSF1 protects against myocardial injury in AMI by suppressing the pro-apoptotic effect of p53 through regulation of *Bcl2L12* splice switching. These findings highlight the significance of SRSF1 in mitigating apoptosis in cardiomyocytes post-myocardial infarction, thus providing valuable insights for potential therapeutic interventions.

## Materials and methods

### Mouse model of acute myocardial infarction (AMI)

Healthy male C57BL/6 mice ranging from 8 to 10 weeks old (weighing 21–25 g) were provided by Guangzhou Laboratory Animal Center (Guangzhou, China). Animals were anesthetized by gas with isoflurane (Reward, China). After connecting mice with an animal ventilator (Reward, Shenzhen, China) by endotracheal intubation, the thoracic cavity was opened between the ribs 3–4 on the left side of the mice, and the left descending coronary artery (LAD) was ligated with a 7/0 nylon suture at 2 mm below the border between left atrium and ventricle. The obvious elevation of S-T segment of electrocardiogram (ECG) indicated successful establishment of AMI model. The sham-operated mice received the same procedure without ligated. The animals were euthanized by cervical dislocation at 24 h after AMI for subsequent experiments. The use of laboratory animals was followed the Guidelines for the Guide for the Care and Use of Laboratory Animals published by the US National Institutes of Health (publication No. 85 − 23; revised, 1996).

**Fig. 1:**
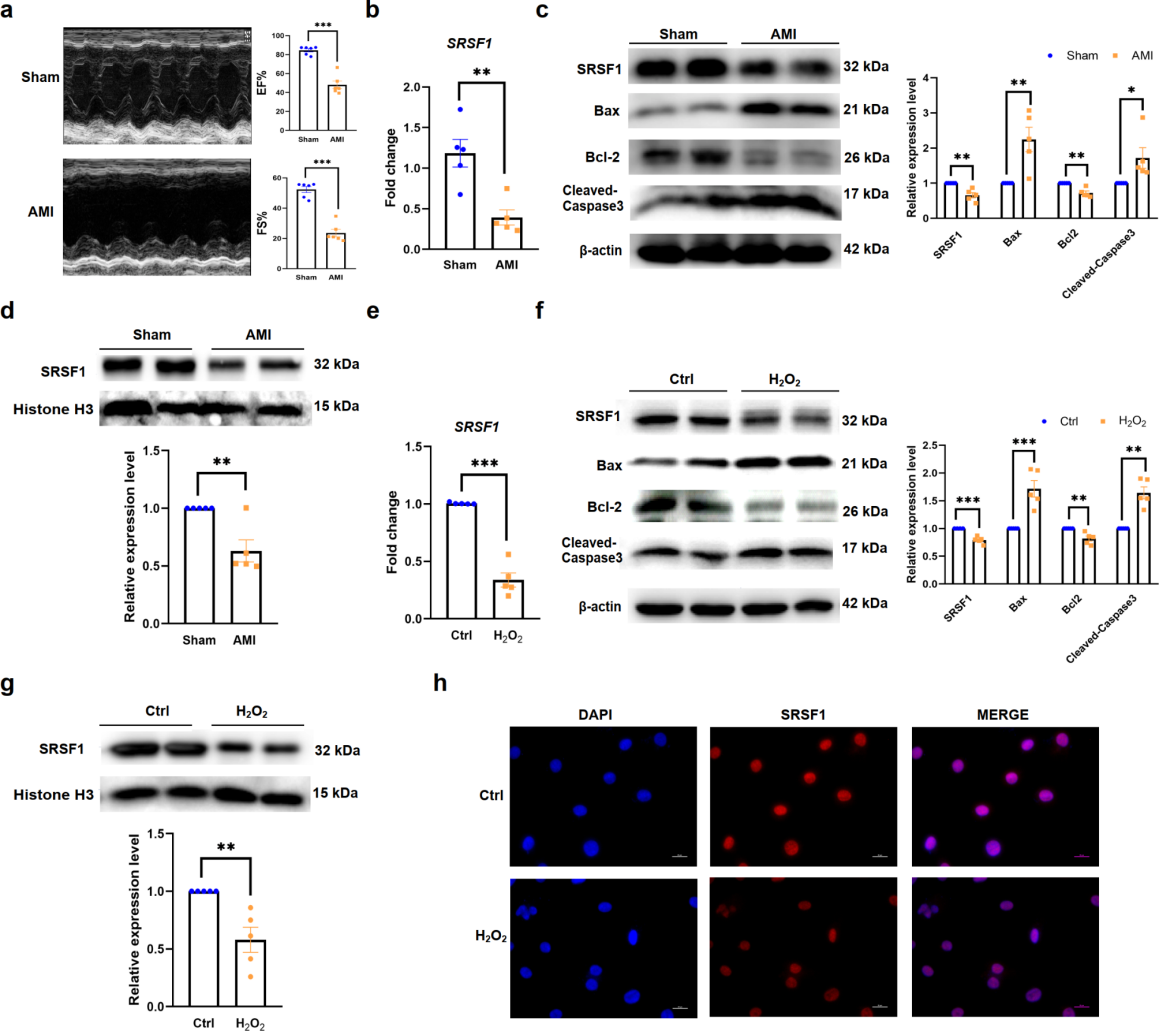
Reduced expression of SRSF1 in AMI mice hearts and H_2_O_2_-treated H9C2 cells. **a**. Echocardiography of AMI model mice (*n* = 6). **b.** qRT-PCR analysis showing the decreased mRNA level of SRSF1 in AMI mice hearts (*n* = 5). **c.** Western blot analyses of SRSF1, Bax, Bcl2 and Cleaved-Caspase3 protein levels in AMI mice hearts (*n* = 5). **d.** Western blot analyses of SRSF1 protein level in nucleus of AMI mice heart tissues (*n* = 5). **e-f.** qRT-PCR analysis of SRSF1 mRNA level and western blot analyses of SRSF1, Bax, Bcl2 and Cleaved-Caspase3 protein levels in H9C2 cells treated by H_2_O_2_ (200 µM) (*n* = 5). **g.** Western blot analysis of SRSF1 protein level in nucleus of H9C2 cells treated with H_2_O_2_ (*n* = 5). **h.** Immunofluorescence (IF) staining showing the protein level of SRSF1 in H9C2 cell nucleus treated with H_2_O_2_ (scale bar = 20 μm, *n* = 5). β-actin and Histone H3 were used as internal controls. Data are expressed as mean ± SEM; *P < 0.05; **P < 0.01; ***P < 0.001

### Construction of adeno-associated virus carrying SRSF1

The adeno-associated virus serotype 9 (AAV9) vector carrying SRSF1 gene was constructed by Genechem (Shanghai, China), and the empty AAV9 vector was used as a control. Mice were injected with AAV9 virus (1 × 10^11^ titer) by tail vein injection (0.15 ml/animal). AMI model was established three weeks after virus injection.

### Echocardiographic measurements

Transthoracic echocardiography was utilized to assess the function of left ventricular (LV) by ultrasound machine Vevo2100 high-resolution imaging system (Visual Sonics, Toronto, Canada) equipped with a 10-MH 2 phased-array transducer with the M-mode recording. The left ventricular parameters including left ventricular end diastolic volume (LVEDV), left ventricular end systolic volume (LVESV), left ventricular internal dimension at end diastole (LVIDd) and left ventricular internal dimension at systole (LVIDs) were measured to analyse left ventricular ejection fraction (EF) and fractional shortening (FS).

**Fig. 2:**
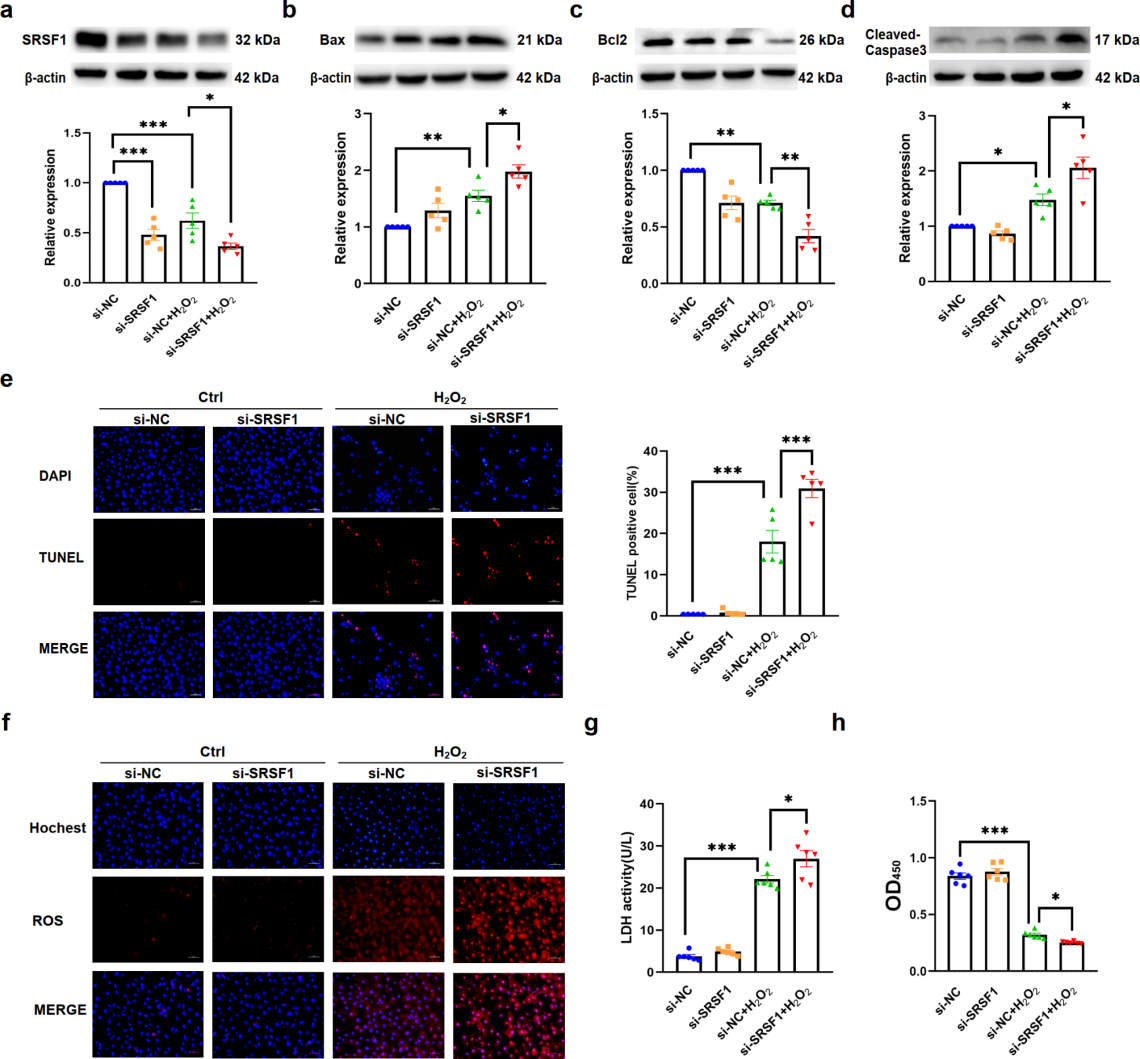
Effect of SRSF1 deletion on H9C2 cells treated with H_2_O_2_. **a-d.** Western blot showing the protein levels of SRSF1, Bax, Bcl2, and Cleaved-Caspase3 in SRSF1 knockdown cells after H_2_O_2_ treatment. *n* = 5. **e.** TUNEL staining assay demonstrating cell apoptosis in SRSF1 knockdown cells after H_2_O_2_ treatment (scale bar = 50 μm, *n* = 5). **f.** The level of mt-ROS after H_2_O_2_ treatment in SRSF1 knockdown cells. (scale bar = 50 μm, *n* = 5). **g.** The activities of lactate dehydrogenase (LDH) in SRSF1 knockdown cells treated by H_2_O_2_ (*n* = 6). **h.** Cell viability measured by CCK8 assay in SRSF1 knockdown cells after H_2_O_2_ treatment (*n* = 6). β-actin was used as an internal control. Data are expressed as mean ± SEM; *P < 0.05; **P < 0.01; ***P < 0.001

### Triphenyl tetrazolium chloride (TTC) staining

The hearts were quickly removed, gently squeezed in PBS to drain the blood, slightly frozen and cut into five pieces. 2.0% TTC (Solarbio, Beijing, China) was used to stain slices within the dark at 37 °C for 20 min, and then immersed in 4% paraformaldehyde for 30 min for fixation. Photographs were taken for analysis, the infarcted area was shown in white and the normal area was shown in red. Image J was utilized to analyze the proportion of infarcted area in the total area.

### Cell culture and treatment

H9C2 cells (Procell, Wuhan, China) were cultured in DMEM (Gibco, California, USA) supplemented with 10% fetal bovine serum (ExcellBio, Shanghai, China) and 1% penicillin-streptomycin (Biosharp, Anhui, China). Cells grown in a humid atmosphere of 5% CO_2_ and 95% air at 37℃. The cell transfection experiment was carried out when the cell confluence reached 70–80%, and the apoptosis model was induced by hydrogen peroxide (200 µM) treatment for 12 h.

**Fig. 3:**
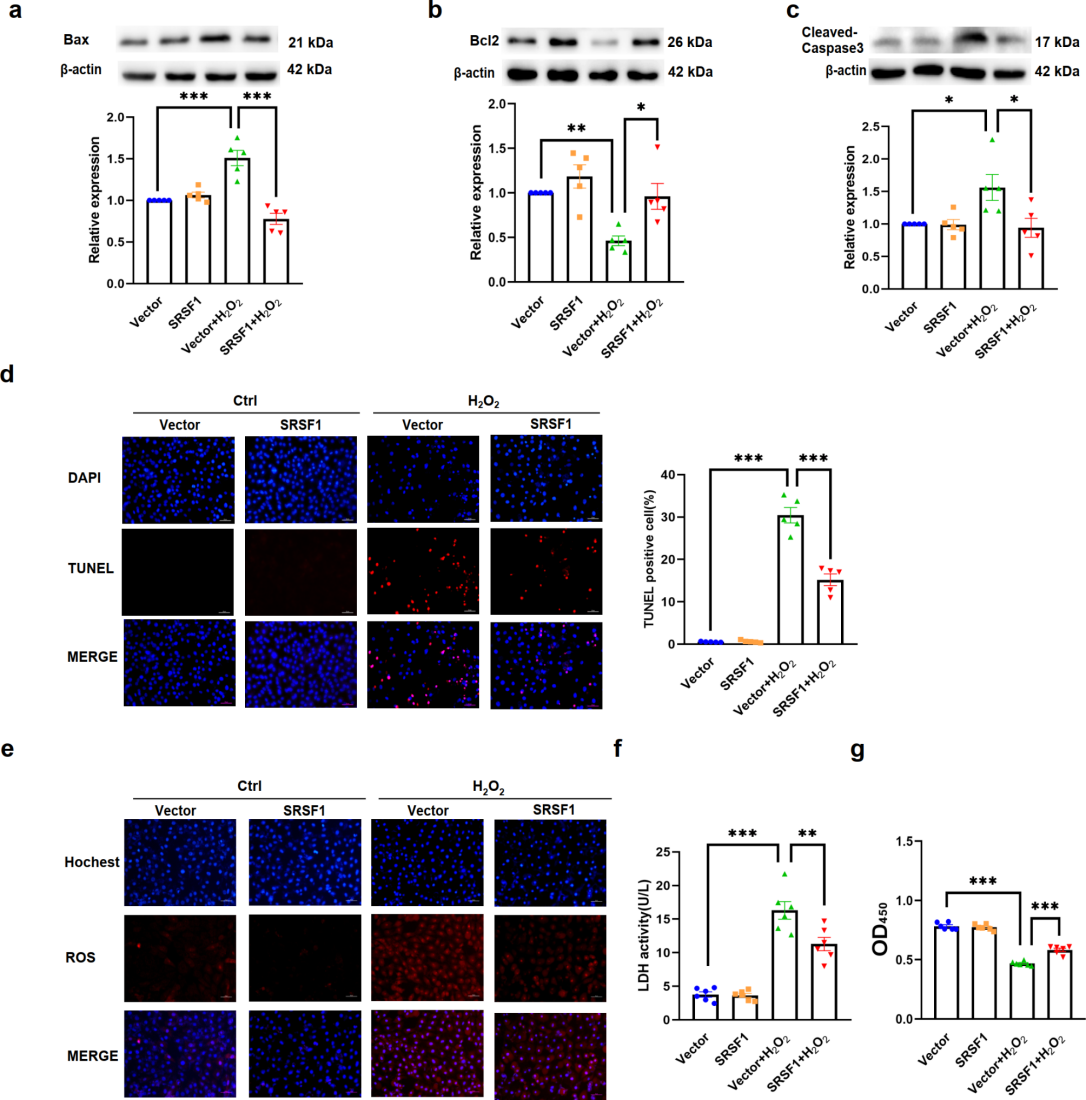
Over-expression of SRSF1 alleviates H_2_O_2_-induced apoptosis in H9C2 cells. **a-c.** Western blot analyses showing the protein levels of Bax, Bcl2, and Cleaved-Caspase3 in SRSF1 overexpression cells after H_2_O_2_ treatment (*n* = 5). **d**. TUNEL staining demonstrating reduced apoptosis in SRSF1 overexpression cells after H_2_O_2_ treatment (scale bar = 50 μm, *n* = 5). **e.** The level of mt-ROS in SRSF1 overexpression cells after H_2_O_2_ treatment. (scale bar = 50 μm, *n* = 5). **f.** The decreased LDH activity in H9C2 cells transfected with SRSF1 overexpression plasmid compared to vector-transfected cells after H_2_O_2_ treatment (*n* = 6). **g.** Cell viability measured by CCK8 assay in SRSF1 overexpression cells after H_2_O_2_ treatment (*n* = 6). β-actin was used as an internal control. Data are expressed as mean ± SEM; *P < 0.05; **P < 0.01; ***P < 0.001

### Synthesis of over-expression plasmids and siRNAs

The siRNAs of SRSF1, Bcl2L12(t) and Bcl2L12(f) were synthesized by GenePharma (Jiangsu, China), and their meaningless sequences were used as control (NC). The plasmids expressing EGFP-fused SRSF1, Flag-fused fulllength Bcl2L12 (Bcl2L12(f)) and truncated Bcl2L12 (Bcl2L12(t)) were constructed using the pcDNA3.1 vector. The empty vector was used as a negative control (Genechem, Shanghai, China). Cells were transfected in siRNAs and plasmids by using Lipofectamine™ 2000 reagent (Invitrogen, CA, USA) and TransIntroTM PL Transfection Reagent (Transgen, Beijing, China), respectively, according to the instructions. The specific sequences are listed in Supplementary Table 1.

### RNA-Seq and data analysis

Total RNA was extracted from H9C2 cells transfected with si-NC and si-SRSF1. The Illumina HiSeq 2000 system was used to RNA-seq pairing according to the instructions. As mentioned previously, the distinct splicing events between samples can be found by read mapping and data analysis [19].

**Fig. 4:**
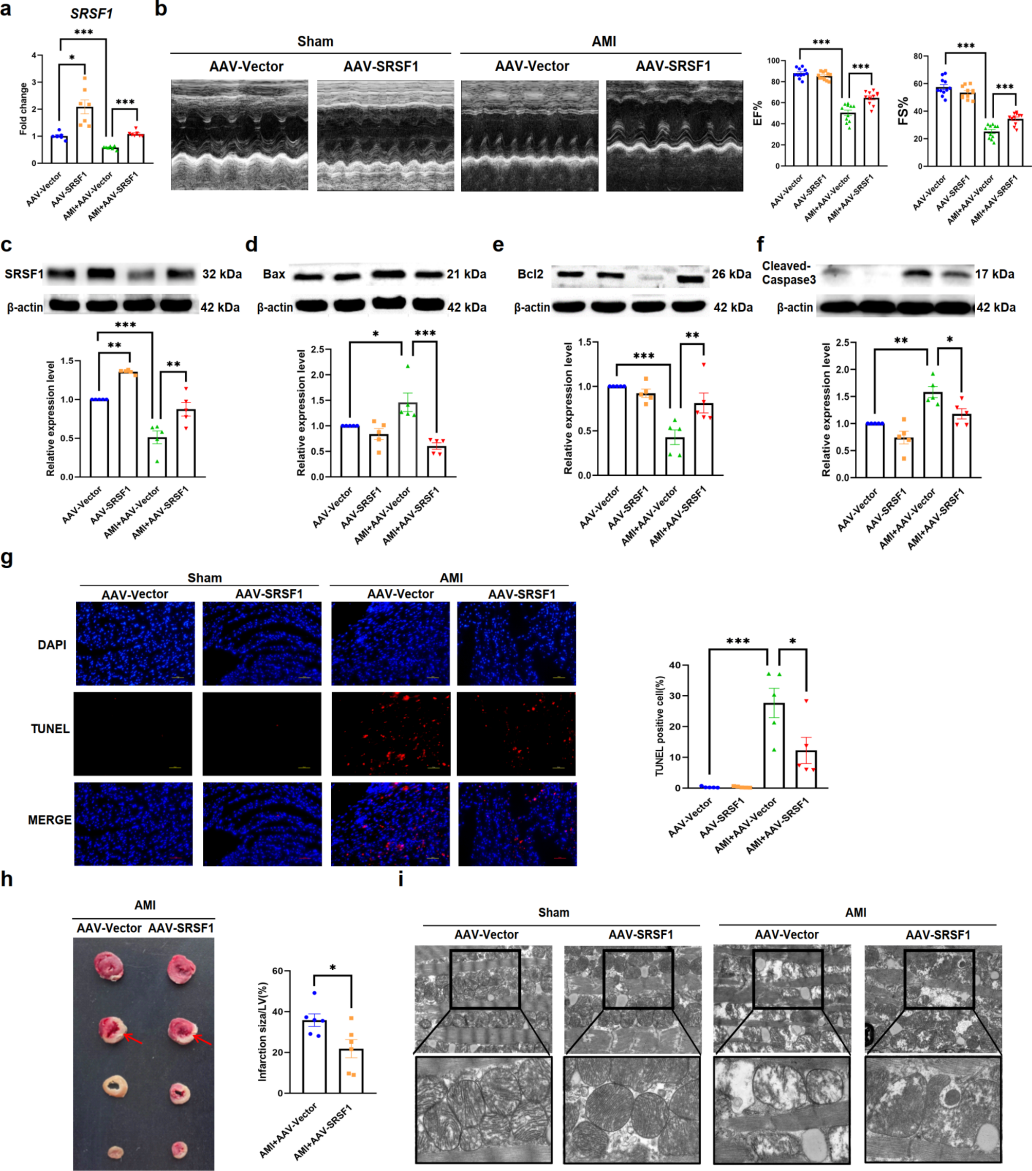
Over-expression of SRSF1 improves cardiac function and alleviates apoptotic damage in AMI mice. **a**. qRT-PCR analysis of SRSF1 mRNA level in AAV-SRSF1-infected AMI mice (*n* = 7). **b.** The cardiac function of AAV-SRSF1-infected AMI mice, detected by echocardiography from the parasternal short axis section. (*n* = 11). **c-f.** Western blot analyses of SRSF1, Bax, Bcl2, and Cleaved-Caspase3 protein levels in AMI mice hearts over-expressing SRSF1 (*n* = 5). **g.** AAV-SRSF1 overexpression reduces the number of TUNEL staining positive cells in AMI mice hearts (scale bar = 50 μm, *n* = 5). **h.** Representative images of TTC staining in AMI mice hearts infected with AAV-SRSF1, the red arrow indicates the position of the tie line (*n* = 6). **i.** AAV-SRSF1 overexpression restores the mitochondrial damage in the hearts of AMI mice (scale bar = 2.0 μm and 500 nm, respectively, *n* = 3). β-actin was used as an internal control. Data are expressed as mean ± SEM; *P < 0.05; **P < 0.01; ***P < 0.001

### Binding motif analysis of SRSF1

We first integrated the results of the online bioinformatic tool ESEfifinder 3.0 (http://krainer01.cshl.edu/cgi-bin/tools/ESE3/esefifinder.cgi?process=home) and Protein-RNA Interaction Predictor (http://bclab.inha.ac.kr/pridictor/pridictor.html) to predict the binding sites of SRSF1 in *Bcl2L12* gene. Besides, we defined GA-rich 6-mers that contained at least one G and one A with GA content ≥ 50% as potential SRSF1-binding sites according to the previously described CLIP analysis results [20], and the degenerate nature of RNA-binding sequences. Finally, three predicted sites were selected to construct sites-mutated minigene for subsequent RNA-protein binding experiments.

**Fig. 5:**
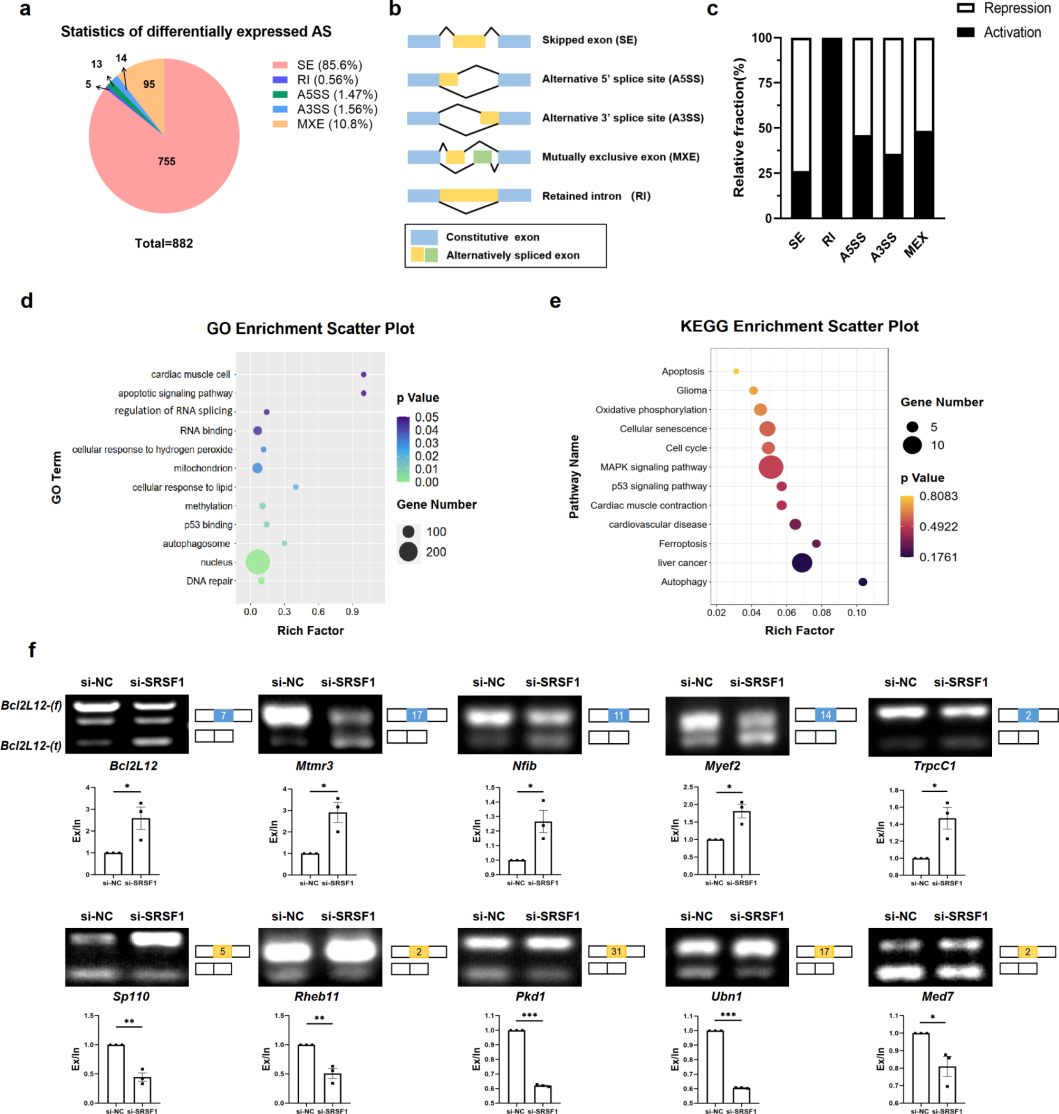
Validation of SRSF1-mediated Alternative splicing (AS) in H9C2 cells. **a-b.** RNA-seq analysis of AS types and amounts occurring in H9C2 cells after SRSF1 knockdown. **c.** The ratio of activation and repression of different splicing types regulated by SRSF1 in H9C2 cells. **d-e.** GO and KEGG enrichment analyses of genes with AS changes after SRSF1 knockdown. **f.** Validation of representative genes with AS changes regulated by SRSF1 in RNA-seq results using RT-PCR and gel electrophoresis. The quantification of RNA products is expressed as exclusion/inclusion (Ex/In). SRSF1 knockout mediated exon exclusion is marked in blue, whereas inclusion is marked in yellow (*n* = 3). Data are expressed as mean ± SEM; *P < 0.05; **P < 0.01; ***P < 0.001

### Minigene reporter assay

Briefly, we inserted the sequence containing *Bcl2L12* exon 6-7-8 and *Bcl2L12* exon 6–7(Mut)-8 into the pcDNA 3.1 vector to construct the minigenes of Bcl2L12 (WT) and Bcl2L12 (Mut), respectively (Cyagen, Suzhou, China). Detailed primer sequences for mingenes are shown in Supplemental Table 3.

### RNA binding protein immunoprecipitation (RIP)

H9C2 cells were cultured in two T75 dishes per sample. RIP experiments were performed according to Magna RIP kit’s instructions (Millipore, Massachusetts, USA). After the cells were washed with pre-cooled PBS, they were scraped from the culture dish with lysate. The treated magnetic beads were combined with SRSF1 antibody (Proteintech, Chicago, USA) and IgG antibody at room temperature, respectively. Next, add the cell lysate to the magnetic bead-antibody, and rotate it at 4°C overnight for RIP immunoprecipitation. The second day, remove the protein from the immune sediment to obtain purified RNA for subsequent RT-PCR verification. Primer sequences used for RNA-RIP are listed in Supplementary Table 3.

**Fig. 6:**
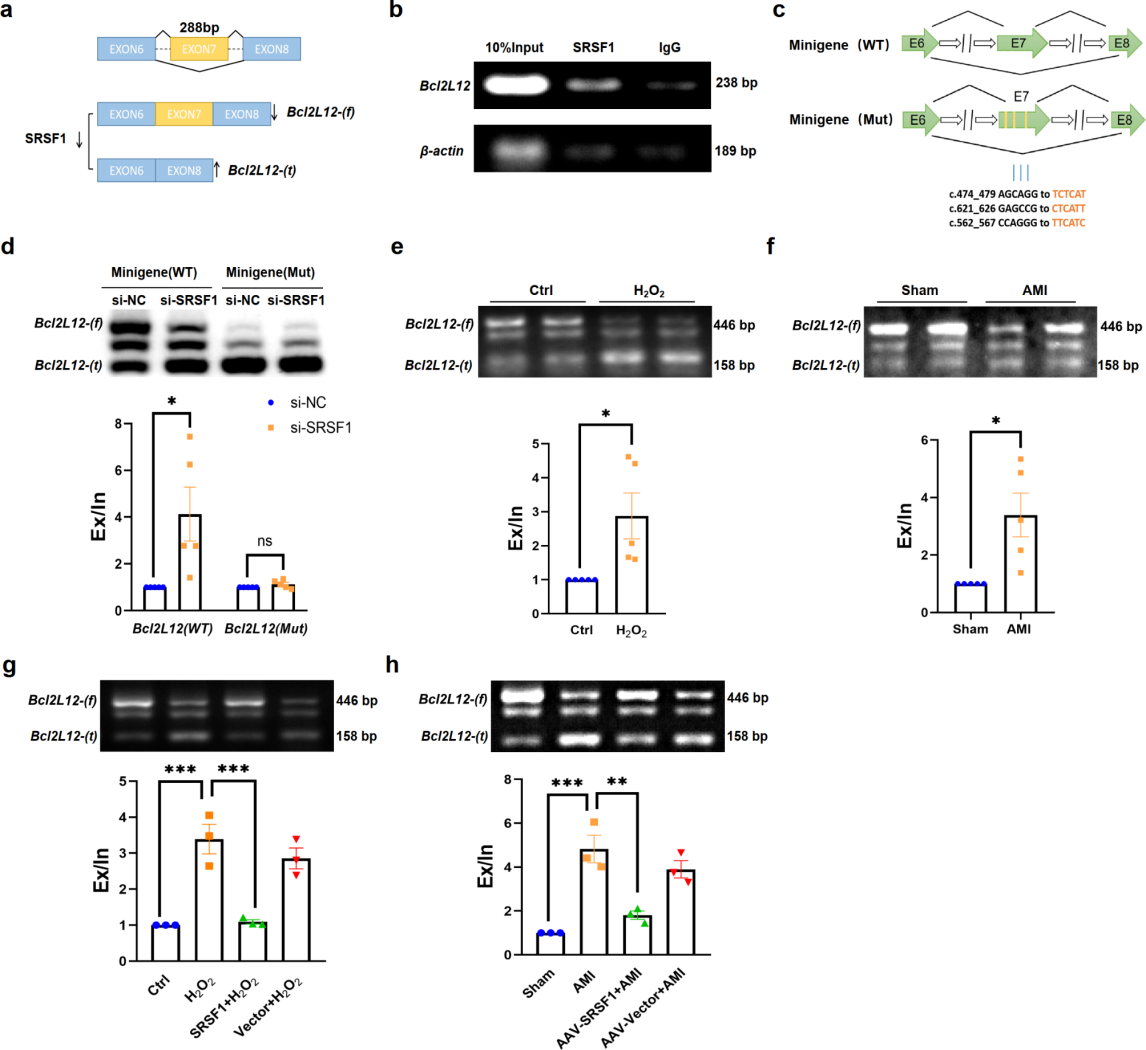
SRSF1 regulates exon7 inclusion by directly binding to *Bcl2L12*. **a**. Schematic diagram illustrating the role of SRSF1 in regulating the splice switching of *Bcl2L12*. *Bcl2L12(t)* represents the truncated subtype of *Bcl2L12*, and *Bcl2L12(f)* represents the full-length subtype of *Bcl2L12*. **b.** Immunoprecipitation of exon7 of *Bcl2L12* using SRSF1 antibody in H9C2 cells (*n* = 3). **c.** Construction of minigene (WT) (*Bcl2L12* exon 6-7-8) and minigene (Mut) (*Bcl2L12* exon 6–7(Mut)-8), with yellow indicating the three mutated sites. **d.** The regulatory effect of SRSF1 knockdown on minigene (WT) and minigene (Mut) (*n* = 5). **e-f.** Increased RNA levels of *Bcl2L12(t)* in H_2_O_2_-induced H9C2 cells and AMI mice hearts (*n* = 5). **g-h.** Overexpression of SRSF1 ameliorated *Bcl2L12* splicing dysregulation in the heart of AMI mice and in H9C2 cells induced by H_2_O_2_ (*n* = 3). Data are expressed as mean ± SEM; *P < 0.05; **P < 0.01; ***P < 0.001

### Co-immunoprecipitation (CO-IP)

Plasmids with Flag-Bcl2L12(f), Flag-Bcl2L12(t), and empty vector were transfected into H9C2 cells, respectively. After 48 h, cells were harvested for CO-IP experiments. After cleaning with pre-cooled PBS, NP-40 (Beyotime, Shanghai, China) lysate was used to scrape cell off the petri dish, and 10% lysate was taken from each sample as input. The cell lysate was combined with the pre-treated BeyoMag™ Anti-Flag Magnetic beads (Beyotime, Shanghai, China) at 4℃ overnight for immunoprecipitation. After repeated washing with TBS, proteins were separated by SDS-PAGE sampling buffer elution method. Finally, western blot assay was used to detect the immunoprecipitate of p53.

**Fig. 7:**
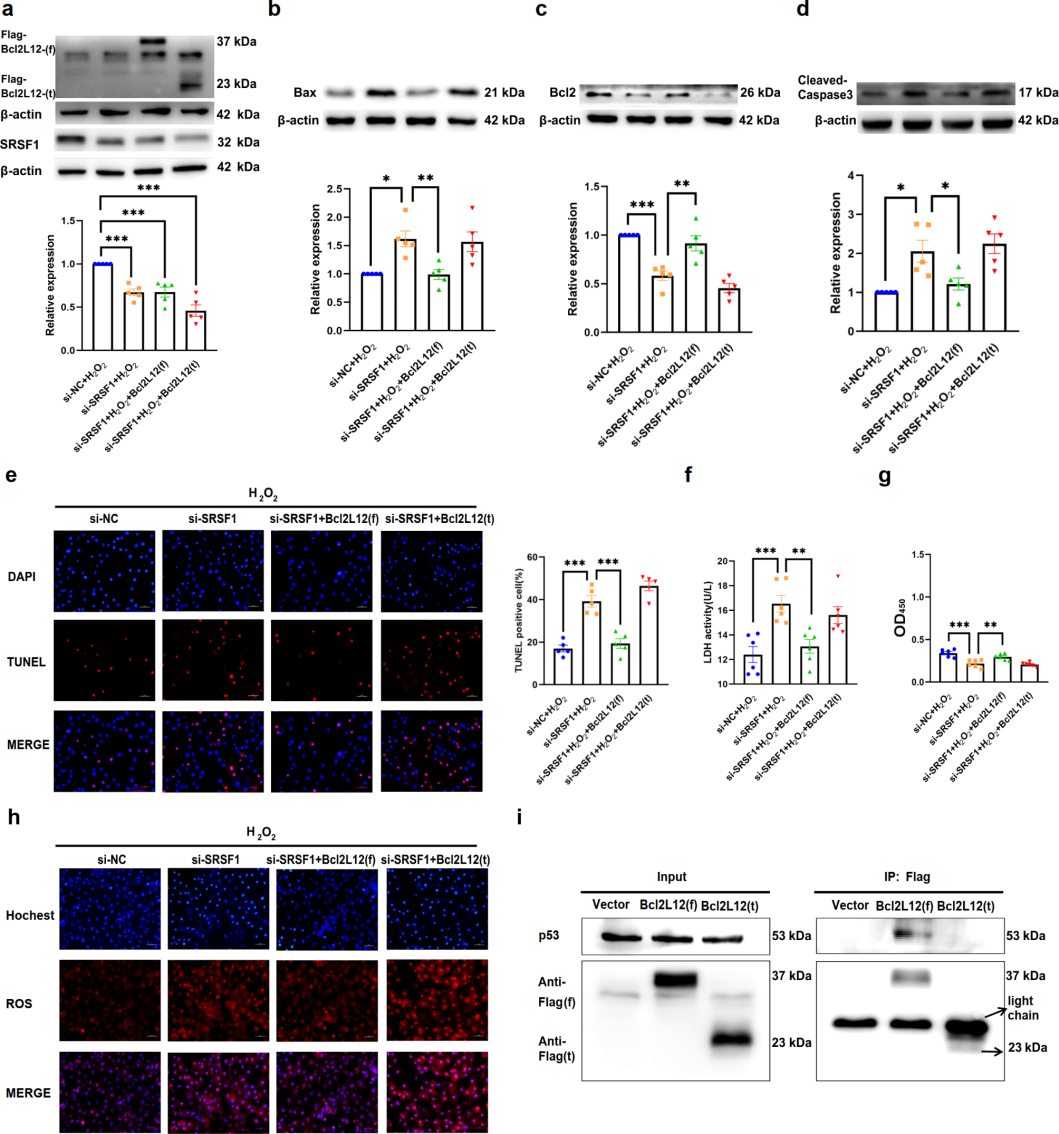
The effects of the Bcl2L12 (f) and Bcl2L12 (t) on SRSF1 knockout cells. **a-d.** The protein levels of SRSF1, Bax, Bcl2, and Cleaved-Caspase3 in H9C2 cells co-transfected with si-SRSF1 and Bcl2L12(f)/Bcl2L12(t) overexpression plasmids after H_2_O_2_ treatment (*n* = 5). **e.** TUNEL staining assay in H9C2 cells co-transfected with si-SRSF1 and Bcl2L12(f)/Bcl2L12(t) in H_2_O_2_ treatment cells (scale bar = 50 μm, *n* = 5). **f.** The LDH level in H_2_O_2_-treated cells co-transfected with si-SRSF1 and Bcl2L12(f)/Bcl2L12(t) (*n* = 6). **g.** Cell viability in H_2_O_2_-treated H9C2 cells co-transfected with si-SRSF1 and Bcl2L12 (f)/Bcl2L12 (t), detected by CCK8 (*n* = 6). **h.** The level of mt-ROS after H_2_O_2_ treatment in si-SRSF1 and Bcl2L12(f)/Bcl2L12(t) co-transfection cells. (scale bar = 50 μm, *n* = 5). **i.** The western blot results of p53 immunoprecipitated by Flag-BclL12(f) (Anti-Flag (f)) and Flag-BclL12(t) (Anti-Flag (t)) (*n* = 3). β-actin was used as an internal control. Data are expressed as mean ± SEM; *P < 0.05; **P < 0.01; ***P < 0.001

### Immunofluorescence staining

Cells were successively incubated with 4% paraformaldehyde for 15 min at room temperature for fixation, 0.5% triton-X 100 for 30 min at room temperature for permeability, and 5% BSA for 1 h at room temperature for sealing. Then, after incubating SRSF1 primary antibody (Proteintech, Wuhan, China) overnight at 4℃, the secondary antibody (Goat Anti-Rabbit, abcam, Cambridge, United Kingdom) was incubated at room temperature in dark for 1 h. After washing the slides with PBS, DAPI (Beyotime, Shanghai, China) was used to re-stain the nucleus. Finally, sealed the film with fluorescent anti-quenching agent (Beyotime, Shanghai, China) and taken photos by ECLIPSE Ts2-FL fluorescence microscope (Nikon, Japan) for analysis.

**Fig. 8:**
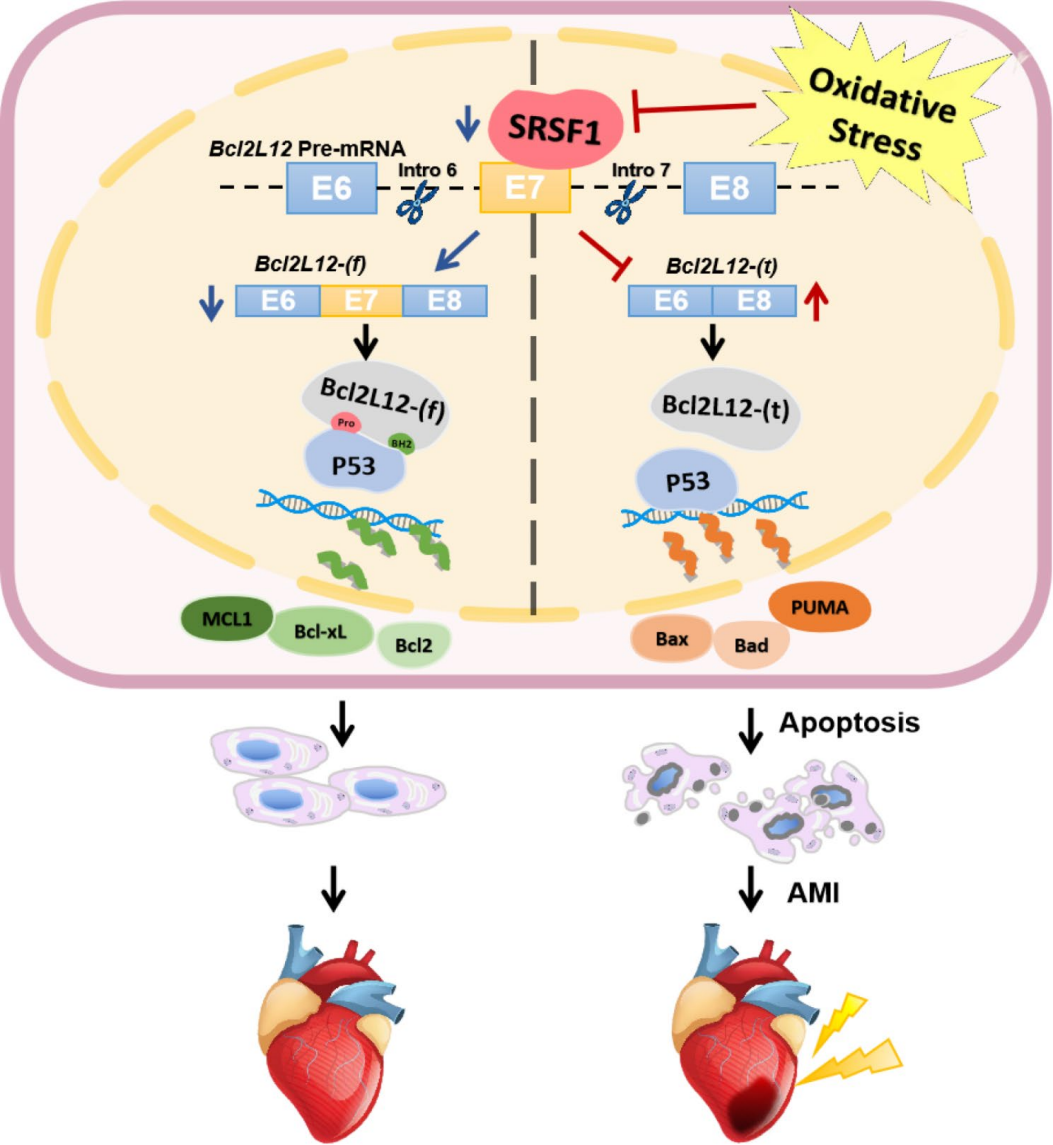
Schematic diagram on the regulation of SRSF1 on cardiomyocyte apoptosis

### TUNEL assay

The In Situ Cell Death Detection Kit, TMR Red (Roche, Basel, Switzerland) was used to detect the apoptosis of paraffin-embedded mice heart tissues and H9C2 cells implanted on the circle microscope cover glass according to the instruction. When positive TUNEL results were observed under fluorescence microscopy, wash the slides with PBS, and then the nuclei were stained with DAPI (Beyotime, Shanghai, China). Photographs were taken using a fluorescence microscope (Nikon, Japan). TUNEL-positive cells and nucleus were counted using Image J to calculate the ratio of TUNEL/DAPI.

### Detection of mitochondrial reactive oxygen species (ROS) in living cells

According to the instructions, MitoSOX™ Red (Invitrogen, California, USA) was used to target superoxides in the mitochondria of H9C2 cells and make them appear red fluorescence. Hochest 33,342 (Solarbio, Beijing, China) was used to stain the nuclei of living cells. Photographs were taken using a fluorescence microscope (Nikon, Japan).

### Western blot analysis

Total protein was extracted from H9C2 cells and mice heart tissues with radio immunoprecipitation assay (RIPA) lysis buffer (Solarbio, Beijing, China), and the protein was quantitatively analyzed by BCA Protein Assay Kit (Beyotime, Shanghai, China). Nuclear protein extraction was performed using a Frozen Sample Nuclear Protein Extraction Kit (Baiaolaibo, Beijing, China). After treatment with SDS-PAGE Sample Loading Buffer (Beyotime, Shanghai, China), proteins with different molecular weights were separated by electrophoresis using 12% SDS polyacrylamide gels. Then, transferred proteins onto nitrocellulose membrane (Pall Corporation, New York, USA). After blocking with 5% skim milk for 1.5 h at room temperature, the first antibody was incubated overnight at 4° temperature: anti-β-actin (Proteintech, Wuhan, China), anti-SRSF1 (Proteintech, Wuhan, China), anti-Bax (Proteintech, Wuhan, China), anti-Bcl2 (Wanlei Biotechnology, Shenyang, China) and anti-Cleaved-Caspase3 (Wanlei Biotechnology, Shenyang, China). After the nitrocellulose membrane was cleaned with TBS prepared by 5% Tween, the corresponding secondary antibody was incubated for 1 h at room temperature. After cleaning with TBS-T, imaging was performed using an AI800 (Cytiva, USA). Image J was used to count the gray value of proteins to calculate the relative content.

### RNA isolation, quantitative real-time PCR and RT-PCR

Total RNA was extracted from H9C2 cells and mice heart tissues by TRIzol (Tiangen, Beijing, China). After quantifying the RNA concentration using a microplate reader (BioTek, Richmond, USA), the All-in-one RT Mix with gDNA Remover Kit (Genstar, Beijing, China) was used to reverse transcribe the RNA into the first strand cDNA. Quantitative real-time PCR was performed by the ABI QuantStudio 7 Flex fast Real-Time PCR system (Applied Biosystems, Foster City, USA) using the 2×RealStar Power SYBR qPCR Mix Kit (Genstar, Beijing, China) and the fold changes of the mRNA levels were calculated by the 2^–ΔΔCt^ method. The detailed sequence information is listed in Supplementary Table 2. RT-PCR was performed by a T100 gradient PCR instrument (Biorad, California, USA) using the 2×EasyTaq^®^ PCR SuperMix (+ dye) (TransGen Biotech, Beijing, China). The RT-PCR products were separated by 1% agarose gel electrophoresis. Image J was used to count the gray values of different spliceosomes to calculate their relative contents. The primer pairs were synthesized by Sangon Biotech (Shanghai, China) and the detailed sequence information is listed in Supplementary Table 3.

### Lactate dehydrogenase activity detection assay (LDH)

Cells were planted in 96-well plates, and LDH release was detected by the LDH Assay Kit (Beyotime, Shanghai, China) after corresponding treatment. The absorbance was measured at 490 nm by a microplate reader (BioTek, Richmond, USA).

### CCK8 assay

100 µl cell suspension was seeded in 96-well plates, after transfection and treatment with H_2_O_2_, 10 µl of CCK8 solution (MedChem Express, New Jersey, USA) was added to each well and mixed evenly. Then, the plates were incubated in an incubator for 1–4 h, and the absorbance at 450 nm was determined using a microplate reader (BioTek, Richmond, USA).

### Statistical analysis

Data were expressed as mean ± SEM. The two-tailed t-test was used to detect differentially expressed genes between two groups. Multiple comparisons were performed by one-way analysis of variance (ANOVA) followed by Tukey’s post-hoc multi-comparison test or Bonferroni post hoc test. GraphPad Prism 8.0 software was used for statistical analysis of experimental data, and the difference of *P* < 0.05 was considered significant.

## Results

### Down-regulation of SRSF1 in AMI mice hearts and H9C2 cells treated with H_2_O_2_

To investigate the role of SRSF1 on apoptosis of cardiomyocytes, we established in vitro and in vivo experimental models by treating H9C2 cells with H_2_O_2_ (200 µmol/L) and performing acute myocardial infarction surgery on C57BL/6 mice. The models were validated by detecting cardiac function (Fig. 1a). Our results found that the mRNA and protein levels of SRSF1 were significantly down-regulated in the hearts of AMI mice, accompanied by increased expression of the pro-apoptotic proteins Bax and Cleaved-Caspase3, and decreased expression of the anti-apoptotic protein Bcl2 (Fig. 1b-c). Although SRSF1 is mainly localized in the nucleus, a small fraction of SRSF1 is continuously shuttled between the nucleus and the cytoplasm. Therefore, we further extracted nuclear proteins to detect the changes of SRSF1 in the nucleus of AMI mice hearts, and the results were the same as those for total protein (Fig. 1d). Consistent with the results in vivo, the mRNA and protein levels of SRSF1 were significantly down-regulated in H9C2 cells treated with H_2_O_2_ (Fig. 1e-f). Western blot results and immunofluorescence staining showed the down-regulated of SRSF1 in the nucleus of H9C2 cells treated with H_2_O_2_ (Fig. 1g-h).

### Inhibition of SRSF1 aggravates H_2_O_2_-induced apoptosis in H9C2 cells

To further investigated the relationship between the down-regulation of SRSF1 and cardiomyocytes apoptosis, we utilized siRNA to knockdown SRSF1 expression. Overexpressed plasmids were constructed to verify whether SRSF1 had a protective effect against injury in vitro. The transfection efficiency was confirmed by qRT-PCR, fluorescence detection and western blot assays (Figure S1). Our results showed that knockdown of SRSF1 further increased the expression of Bax and Cleaved-Caspase3, while reducing the expression of Bcl2 in H9C2 cells treated with H_2_O_2_ (Fig. 2a-d). In addition, knockdown of SRSF1 further increased the number of TUNEL-positive cells (Fig. 2e), mitochondrial reactive oxygen species (mt-ROS) levels (Fig. 2f), LDH activity (Fig. 2g), and inhibited cell viability (Fig. 2h).

### Over-expression of SRSF1 alleviates apoptosis of H9C2 cells induced by H_2_O_2_ and attenuates cardiac injury in AMI mice

In contrast, over-expression of SRSF1 attenuated H_2_O_2_-induced apoptosis in H9C2 cells. Western blot results showed that SRSF1 overexpression reversed the increased expression of Bax and Cleaved-Caspase3, while restoring the decreased expression of Bcl2 under H_2_O_2_ stimulation (Fig. 3a-c). Moreover, over-expression of SRSF1 partially reversed the increase in the number of TUNEL-positive cells (Fig. 3d), mt-ROS levels (Fig. 3e), LDH activity (Fig. 3f), and improved cell viability (Fig. 3g) in H_2_O_2_-treated H9C2 cells. Similar results were observed in H9C2 cells subjected to hypoxia. Overexpression of SRSF1 reversed the apoptosis of cardiomyocytes under hypoxia treatment (Figure S2).

To validate our findings in an in vivo setting, we utilized an AAV9 adenovirus vector carrying the SRSF1 gene (AAV-SRSF1) to over-express SRSF1 in mice heart tissues. AMI model was established by ligation of the left anterior descending coronary artery (LAD) three weeks after tail vein injection of AAV-SRSF1. Cardiac function and tissue infarction were assessed 24 h post-surgery. Over-expression of SRSF1 in mice heart tissues was confirmed by qRT-PCR (Fig. 4a). In AMI mice, compared with those injected with AAV-Vector, over-expression of SRSF1 improved cardiac function of AMI mice, as evidenced by increased left ventricular ejection fraction and left ventricular shortening fraction (Fig. 4b). Additionally, compared with the AAV-Vector, over-expression of SRSF1 decreased the protein expression levels of Bax and Cleaved-Caspase3, and increased the protein expression levels of Bcl2 in the hearts of AMI mice (Fig. 4c-f). TUNEL assay showed that SRSF1 reduced the number of apoptotic cells in cardiac tissues of AMI mice (Fig. 4g). The TTC test showed a reduction in the size of myocardial infarction in SRSF1 over-expressed mice (Fig. 4h). Furthermore, electron microscopy assay revealed that the morphology of mitochondria was more complete in cardiomyocytes of mice over-expressing (Fig. 4i). Collectively, these results indicate that SRSF1 exert a protective effect on cardiac injury and inhibits apoptosis in the context of myocardial infarction.

### AS events in H9C2 cells mediated by SRSF1

SR protein family regulates splicing to produce different mRNA isoforms by binding to pre-mRNA, which is the main mechanism of their biological functions. To further explore the mechanism of anti-apoptotic effect of SRSF1 in myocardial cells, we performed high-throughput sequencing of RNA (RNA-seq) on H9C2 cells in the control (NC) group and the SRSF1 knockdown group. As shown in Figure S3a-b, a total of 1 264 genes were altered after SRSF1 knockdown, of which 776 genes were down-regulated and 488 genes were up-regulated. Gene Ontology (GO) and Kyoto Encyclopedia of Genes and Genomes (KEGG) analyses highlighted the close association of the differentially expressed genes with cardiovascular diseases. In addition, they were found to be involved in functions such as apoptosis, glycolysis and protein binding, as well as pathways such as PI3K-AKT and cGMP-PKG (Figure S3c-d).

Subsequently, we focused on the impact of SRSF1 on alternative splicing events and identified genes with significant differences between the NC group and the SRSF1 knockdown group, using criteria of *P* < 0.05 and PSI > 0.1. The data showed that SRSF1 knockdown resulted in a total of 882 abnormal splicing events, including skipped exon (SE), retained intro (RI), alternative 5’splice site (A5SS), alternative 3’splice site (A3SS) and mutually exclusive exon (MEX) splicing types. Among these, 755 events were classified as SE events, accounting for 85.6% of the total (Fig. 5a-b). Notably, SRSF1 exhibited a bidirectional role in splicing activation and repression across all four splicing types except RI (Fig. 5c). Further GO enrichment analysis of genes with abnormal splicing demonstrated a correlation between SRSF1 and functions related to oxidative stress, autophagy, apoptosis, and DNA repair. Additionally, KEGG enrichment analysis indicated that SRSF1 was closely associated with the p53 and MAPK pathway (Fig. 5d-e). These findings provide additional support for the potential involvement of SRSF1 in cardiomyocyte apoptosis.

### SRSF1 directly binds to and regulated the *Bcl2L12* splice switching

To validate the abnormal splicing events identified by RNA-seq, 10 genes that exhibited splicing activation and splicing repression after SRSF1 knockdown were selected for verification using RT-PCR and gel electrophoresis assays (Fig. 5f & Figure S4). Among these genes, particular attention was given to *Bcl2L12*, an apoptosis-related gene. As illustrated in Fig. 6a, *Bcl2L12* splicing was suppressed in SRSF1 knockdown cells, specifically characterized by a significant exon7 splicing event.

Bcl2L12 belongs to the Bcl2 family and is characterized by its BH2 domain and abundant proline content. To further explore the underlying mechanism of SRSF1 on *Bcl2L12* alternative splicing, we performed RNA immunoprecipitation (RIP) experiments targeting exon7 of *Bcl2L12* with SRSF1 antibody. The results confirmed the direct interaction between SRSF1 and exon7 of *Bcl2L12* (Fig. 6b). Then, we utilized the ESEfifinder 3.0 and Protein-RNA Interaction Predictor tools to predict the potential binding sites of SRSF1 on exon7 of *Bcl2L12*. Based on these predictions, we constructed two types of minigenes: a wild-type (WT) subtype and a mutant (Mut). The results showed that the splicing of the WT minigene could still be regulated by SRSF1, whereas the mutated minigene showed minimal changes, confirming the direct binding of SRSF1 to exon7 of *Bcl2L12* (Fig. 6c-d). Furthermore, the full-length isoform of *Bcl2L12* was down-regulated and the truncated isoform was up-regulated in H9C2 cells exposed to H_2_O_2_ (Fig. 6e) and the heart tissues of AMI mice (Fig. 6f), indicating its potential association with apoptosis. The results in Fig. 6g and h confirmed that overexpression of SRSF1 reversed *Bcl2L12* aberrant splicing after damage, both at the in vivo and in vitro levels.

### Effect of different Bcl2L12 isoforms on H9C2 cell damage aggravated by SRSF1 knockdown

To investigate the function differences of Bcl2L12 isoforms, we constructed Flag- labeled fusion overexpression plasmids for the full length of Bcl2L12 (Bcl2L12(f)) and the truncated subtype lacking exon 7 (Bcl2L12(t)). Western Blot results confirmed that Bcl2L12(f) overexpression partially reversed the increased protein expression of Bax and Cleaved-Caspase3 and the decreased protein expression of Bcl2 caused by SRSF1 deletion in H_2_O_2_-induced H9C2 cells (Fig. 7a-d). In contrast, over-expression of Bcl2L12(t) did not exhibit a restorative effect. Consistent with this result, Bcl2L12(f) overexpression reversed the increased number of TUNEL-positive cells (Fig. 7e), the increased LDH activity (Fig. 7f), the decreased cell viability (Fig. 7g), and the elevated mt-ROS expression (Fig. 7h). However, over-expression of Bcl2L12(t) did not demonstrate a recovery effect, indicating the loss of its anti-apoptotic ability.

Furthermore, we constructed siRNA targeting Bcl2L12(t) to further explore its role in apoptosis. The results showed that the knockdown of Bcl2L12(t) exerted a restorative effect on H_2_O_2_-induced apoptosis in H9C2 cells (Figure S5). Conversely, Bcl2L12(t) overexpression further aggravated H_2_O_2_-induced apoptosis in H9C2 cells (Figure S6), suggesting a potential negative feedback regulation between different isoforms of Bcl2L12.

### SRSF1 regulates the binding of Bcl2L12 to p53 by regulating *Bcl2L12* splicing

It has been reported that Bcl2L12 can inhibit the function of p53, a transcription factor in the nucleus, by binding to it, thereby exerting an anti-apoptotic role. We then applied co-immunoprecipitation (CO-IP) assay to investigate the interaction between Bcl2L12 and p53. The results confirmed the binding of Bcl2L12(f) to the p53 protein. In contrast, the truncated subtype of Bcl2L12 did not exhibit significant binding with p53 protein, suggesting a lack of interaction between them (Fig. 7i). Consistent with these results, we found that the protein structure of Bcl2L12(t) lacks the PPSPDP proline ring and BH2 domain, which are likely crucial for its binding ability (Figure S7).

## Discussion

In the present study, we investigated the role of SRSF1 in cardiomyocyte apoptosis. Subsequently, we explored the regulatory function of SRSF1 as a splicing factor on downstream AS events in cardiomyocytes using RNA-seq analysis. We found that SRSF1 can interact with *Bcl2L12* to regulate its splice switching between full-length and exon7 deletion isoforms. Our findings indicate that different isoforms of Bcl2L12 play distinct roles in anti-apoptotic process of cardiomyocytes by interacting with p53. These results deepen our understanding of alternative splicing in cardiomyocyte apoptosis and suggest that SRSF1 may have potential therapeutic implications for mitigating cardiomyocyte apoptosis.

SRSF1, also known as ASF/SF2, is the prototypical member of the SR protein family, initially identified as a regulator of alternative splicing. However, SR proteins exert their influence on various stages of the mRNA life cycle, including transcription initiation and elongation, mRNA stability, translation and decay [21–23]. Aberrant expression of SRSF1 has been reported in various types of tumors, leading to abnormal RNA splicing, which in turn promotes the proliferation, migration and apoptosis resistance of tumor cells [5, 15, 16, 24]. Studies focused on tumor cell apoptosis have revealed that SRSF1 promotes the generation of anti-apoptotic splice variants of Bcl-x and Mcl-1, thereby reducing programmed cell death in breast cancer and chronic myeloid leukemia [25], whereas down-regulation of SRSF1 triggers the splicing of pro-apoptotic isoforms and leading apoptosis [26]. Furthermore, in cardiovascular disease, it has been reported that the exosome LINC00174 attenuates myocardial I/R injury in rats through negative regulation of p53 signaling by SRSF1 [27].

In the present study, we found the downregulation of SRSF1 in the nucleus of cardiomyocytes in AMI mice and H9C2 cells induced by H_2_O_2_, suggesting a potential anti-apoptotic role of SRSF1 in the heart. Although there was a trend towards increased expression of the pro-apoptotic protein Bax and a decreased expression of the anti-apoptotic protein Bcl2 in SRSF1 knockdown cells, the TUNEL assay did not detect positive cells, and the CCK8 assay did not reveal a significant effect of SRSF1 deletion on cell viability. This indicates that changes in SRSF1 do not significantly regulate cardiomyocyte apoptosis under physiological conditions, which is consistent with previous studies [17, 28]. However, in the pathological conditions, our results demonstrate the protective effect of SRSF1 on myocardial injury and cardiomyocyte apoptosis in the infarcted hearts, suggesting that SRSF1 may indirectly regulate cardiomyocyte apoptosis through downstream target genes. In addition, we examined the expression of inflammatory cytokines in cardiac tissues of SRSF1 overexpressing mice. The results showed that SRSF1 also reversed the expression of IL1-β and IL6 in the hearts of AMI mice. Whether the splicing factor SRSF1 similarly regulates inflammatory progression by regulating the splicing of downstream factors may be another interesting topic to investigate.

The mitochondrial pathway is a crucial pathway that regulates cell apoptosis, initiated by intracellular stimuli such as oxidative stress, hypoxia, and nutrient deprivation. During apoptosis, the mitochondrial membrane permeability increases, leading to the release of pro-apoptotic factors (such as Cyt-C and AIF) into the cytoplasm, and triggers the caspase cascade, ultimately resulting in apoptosis [29]. The accumulation of ROS in mitochondria can cause cell damage through oxidative stress and participate in the initiation of cell apoptosis, thereby promoting cell death signaling [30]. Our findings support the inhibitory effect of SRSF1 on mitochondrial ROS accumulation. Additionally, after the injury-induced stimulation of cells by ischemia and hypoxia, mitochondria exhibit fragmented networks and swollen morphology, eventually leading to the formation of vacuoles [31]. In our results, SRSF1 plays a protective role in maintaining mitochondrial morphology.

Although there are several RNA-Seq databases available for exploring SRSF1-related changes in cancer, the knowledge regarding the AS networks regulated by SRSF1 in the heart is still limited. Through RNA-Seq data analysis, we identified a total of 882 differentially AS events in the SRSF1 knockout H9C2 cells. GO and KEGG enrichment analyses revealed the correlation between these differentially spliced genes and signaling pathways involved in apoptosis and p53 regulation.

Among the genes displaying altered AS, we focused on *Bcl2L12*. The Bcl2 family members are characterized by the presence of at least one BH1, BH2, BH3, or BH4 domain, with the BH2 domain predominantly found in anti-apoptotic proteins and the BH3 domain present in pro-apoptotic member [32]. Bcl2L12, localized in both the nucleus and cytoplasm, is an atypical Bcl2 family member with a BH2 domain and abundant proline sites [33]. Studies have demonstrated that Bcl2L12 co-localizes and binds to p53 in the nucleus of gliomas, inhibiting the transcriptional regulation of p53 by interfering with its binding to target genes [34]. In the cytoplasm, Bcl2L12 can inhibit the maturation of mitochondrial effector proteins, such as caspase-3 and caspase-7, thereby exerting anti-apoptotic effects [35–37]. In cardiovascular diseases, Bcl2L12 forms a complex with c-Myc in eosinophils (Eos) to inhibit FasL expression and confer apoptosis resistance of Eos in myocardial tissue [38]. Moreover, as a gene regulated by multiple spliceosomes, the splicing factor BUD31 has been reported to promote the generation of anti-apoptotic isoform of Bcl2L12, containing exon3, and contribute to the progression of ovarian cancer [39].

Nevertheless, the studies focusing on Bcl2L12 and its spliceosome in the heart are still limited. In this study, through RIP analysis, we identified and confirmed the binding of SRSF1 to *Bcl2L12*, promoting the generation of the full-length isoform of *Bcl2L12* that includes exon7. To further validate the binding of SRSF1 to *Bcl2L12* exon7, we first defined GA-rich 6-mers containing at least one G and one A with ≥ 50% GA content as potential SRSF1 binding sites based on previous findings [20]. Subsequently, we utilized ESEfifinder 3.0 and Protein-RNA Interaction Predictor software to predict the binding sequence, integrating the results from both software tools. Finally, three sequences were selected for simultaneous mutation to construct a minigene. The results from the mingene supported our hypothesis. It is noteworthy that three bands appeared in our gel electrophoresis results, and then we recovered the RT-PCR electrophoresis products and subjected the three bands to DNA sequencing. The sequencing results revealed multiple mutated A-set peaks in the intermediate product, indicating that the product was a non-specific band generated by the secondary structure of the template sequence and unrelated to the target band. The first and third bands respresented the full-length *Bcl2L12* subtype and the exon7 deletion subtype, respectively.

In subsequent studies, we further elucidated the functions of full-length and exon7 deletion truncated isoforms of Bcl2L12, and found that these isoforms exerted anti-apoptotic and pro-apoptotic roles in cardiomyocytes, respectively. P53 promotes apoptosis by regulating the transcription of apoptotic factors, which is one of the important mechanisms underlying the nuclear initiation pathway of cell apoptosis. As reported in previous studies, activated p53 was found to mediate transcriptional activation of a variety of mitochondrial pathway apoptotic proteins such as Puma, Bad and Bax [40]. In addition, p53 also inhibits the transcriptional expression of anti-apoptotic factors such as Bcl2, Bcl-xL and Mcl1 [41–43]. The CO-IP assay confirmed the binding of p53 to the full-length isoform of Bcl2L12 but not the exon7 deletion isoform. Due to the inclusion of the proline loop and BH2 domain, inhibition of protein activity through protein interaction is an important way for Bcl2L12 to function.

However, there are some limitations in our research. Firstly, the process of cell apoptosis is extremely complex, and Bcl2L12 may not be the only downstream gene involved in SRSF1-mediated regulation of cardiomyocyte apoptosis. Secondly, our experimental models were limited to rats and mice. Although SRSF1 and Bcl2L12 exhibit good conservatism, further exploration is required to understanding their functions in the human heart.

## Conclusion

In summary, our findings suggested that SRSF1 may mediate the function of p53 through regulating the splicing of *Bcl2L12*, thereby playing an anti-apoptotic role in cardiomyocytes (Fig. 8). This study emphasizes the importance of alternative splicing in cardiovascular diseases and suggests the therapeutic potential of SRSF1 for cardiomyocytes apoptosis and myocardial infarction.

## Electronic supplementary material

Below is the link to the electronic supplementary material.

Supplementary Material 1

Supplementary Material 2

## Data Availability

All data that support the findings of this study are available from the corresponding author upon reasonable request.

## Abbreviations

AS: Alternative splicing
SRSF1: Serine/arginine splicing factor 1
AMI: Acute myocardial infarction
HF: Heart failure
RRM: Recognition motif
AAV9: deno-associated virus serotype 9
EF: Ejection fraction
FS: Fractional shortening
WT: Wild type
Mut: Mutant
RIP: RNA binding protein immunoprecipitation
CO-IP: Co-Immunoprecipitation
ROS: Reactive oxygen species
PCR: polymerase chain reaction
LDH: Lactate dehydrogenase
RNA-seq: High-throughput sequencing of RNA
GO: Gene Ontology
KEGG: Kyoto Encyclopedia of Genes and Genomes
