# Supplementary material for: Splicing factor SRSF1 attenuates cardiomyocytes apoptosis via regulating alternative splicing of Bcl2L12
Source: Cell Biosci. 2024 Nov 22;14:142. doi: 10.1186/s13578-024-01324-3 (PMC11585136; doi:10.1186/s13578-024-01324-3)
Supplement: Supplementary file 1 — Supplementary Material 1 [file 13578_2024_1324_MOESM1_ESM.docx]

**Supplementary Material**

**Splicing factor SRSF1 attenuates cardiomyocytes apoptosis via regulates alternative splicing of *Bcl2L12***

Yilin Xie^1^^,3&^, Zhenbo Yang^5&^, Wenxian Chen^5^, Changsheng Zhong^5^, Mengyang Li^5^, Lei Zhang^5^, Ting Cheng^5^, Qin Deng^6^, Huifang Wang^5^, Jin Ju^5^, Zhimin Du^1,2*^, Haihai Liang^1,4,7*^

^1^Zhuhai People's Hospital, Guangdong Provincial Key Laboratory of Tumor Interventional Diagnosis and Treatment, Zhuhai Hospital Affiliated With Jinan University, Jinan University, Zhuhai, 519000, Guangdong, China.

^2^State Key Laboratory of Quality Research in Chinese Medicines, Macau University of Science and Technology, Macau 999078, China

^3^Department of Pharmacy, The Third Affiliated Hospital (The Affiliated Luohu Hospital) of Shenzhen University, Shenzhen, Guangdong, 518000, China

^4^State Key Laboratory of Frigid Zone Cardiovascular Diseases (SKLFZCD), Department of Pharmacology (State Key Labratoray -Province Key Laboratories of Biomedicine-Pharmaceutics of China, Key Laboratory of Cardiovascular Research, Ministry of Education), College of Pharmacy, Harbin Medical University, Harbin, 150081, China.

^5^College of Pharmacy, Shenzhen University Medical School, Shenzhen University, Shenzhen, Guangdong, 518055, China

^6^College of Basic Medical Sciences, Shenzhen University Medical School, Shenzhen University, Shenzhen, Guangdong, 518055, China

^7^Research Unit of Noninfectious Chronic Diseases in Frigid Zone (2019RU070), Chinese Academy of Medical Sciences, Harbin, 150081, China.

^&^ With equal contributions to the work

^*^Corresponding to Prof. Haihai Liang ([lianghaihai@ems.hrbmu.edu.cn](mailto:lianghaihai@ems.hrbmu.edu.cn)) or Prof. Zhimin Du ([dzm1956@126.com](mailto:dzm1956@126.com)).

**
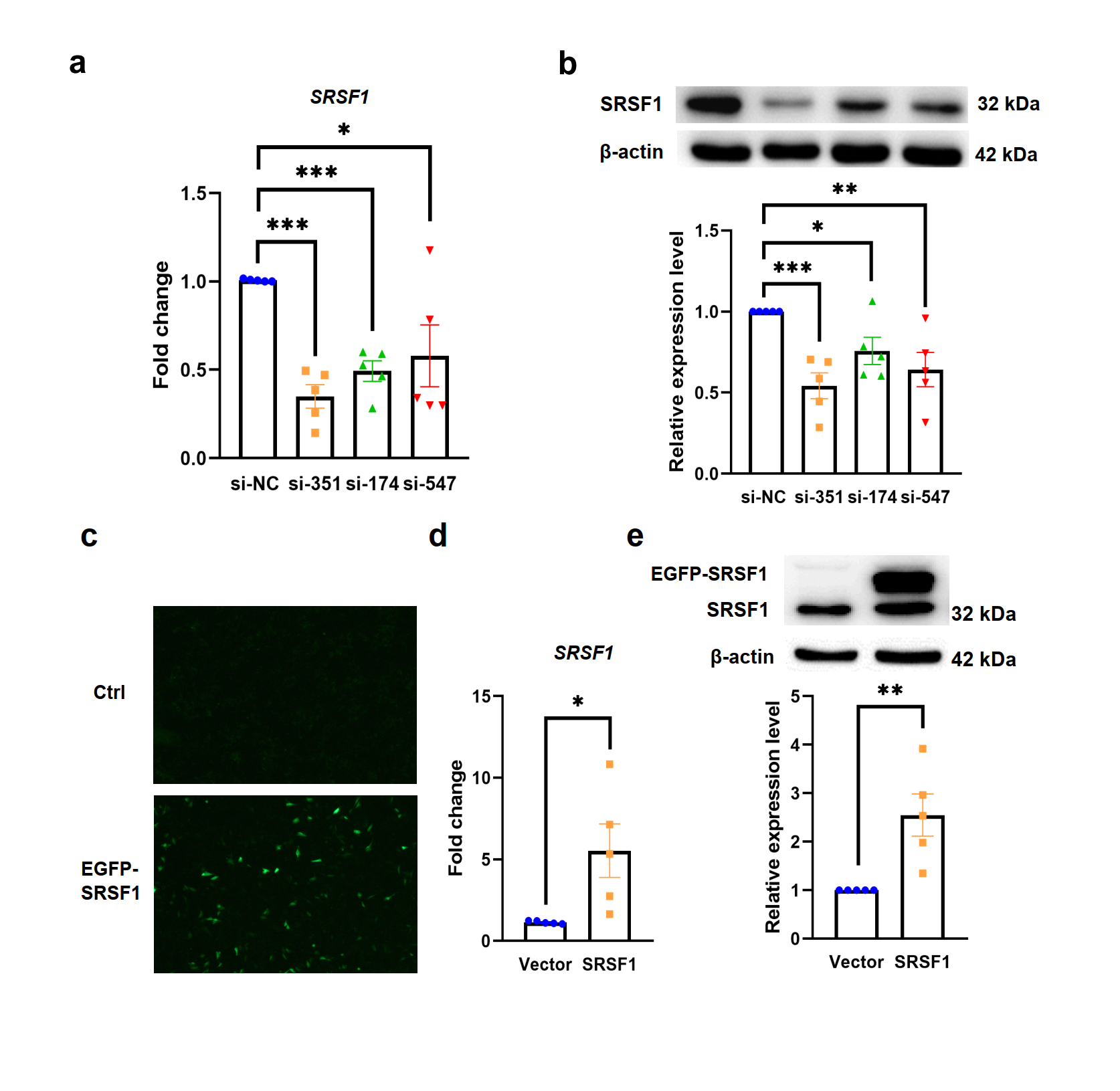
**

**Figure S1. Validation of transfection efficiency of SRSF1 siRNA and over-expression plasmids.**

1. **b.** Detection of mRNA and protein levels of SRSF1 in H9C2 cells transfected with three si-SRSF1. Among them, si-351 was selected for subsequent experiments (n=5). **c-d.** Evaluation of cell transfection efficiency with EGFP-SRSF1 overexpressed plasmid through fluorescence detection and qRT-PCR analysis of SRSF1 mRNA level (n=5). **e.** The protein levels of SRSF1 transfected SRSF1 overexpression plasmids (n=5). β-actin was used as an internal control. Data are expressed as mean ± SEM; **P* < 0.05; ***P* < 0.01; ****P* < 0.001.

**
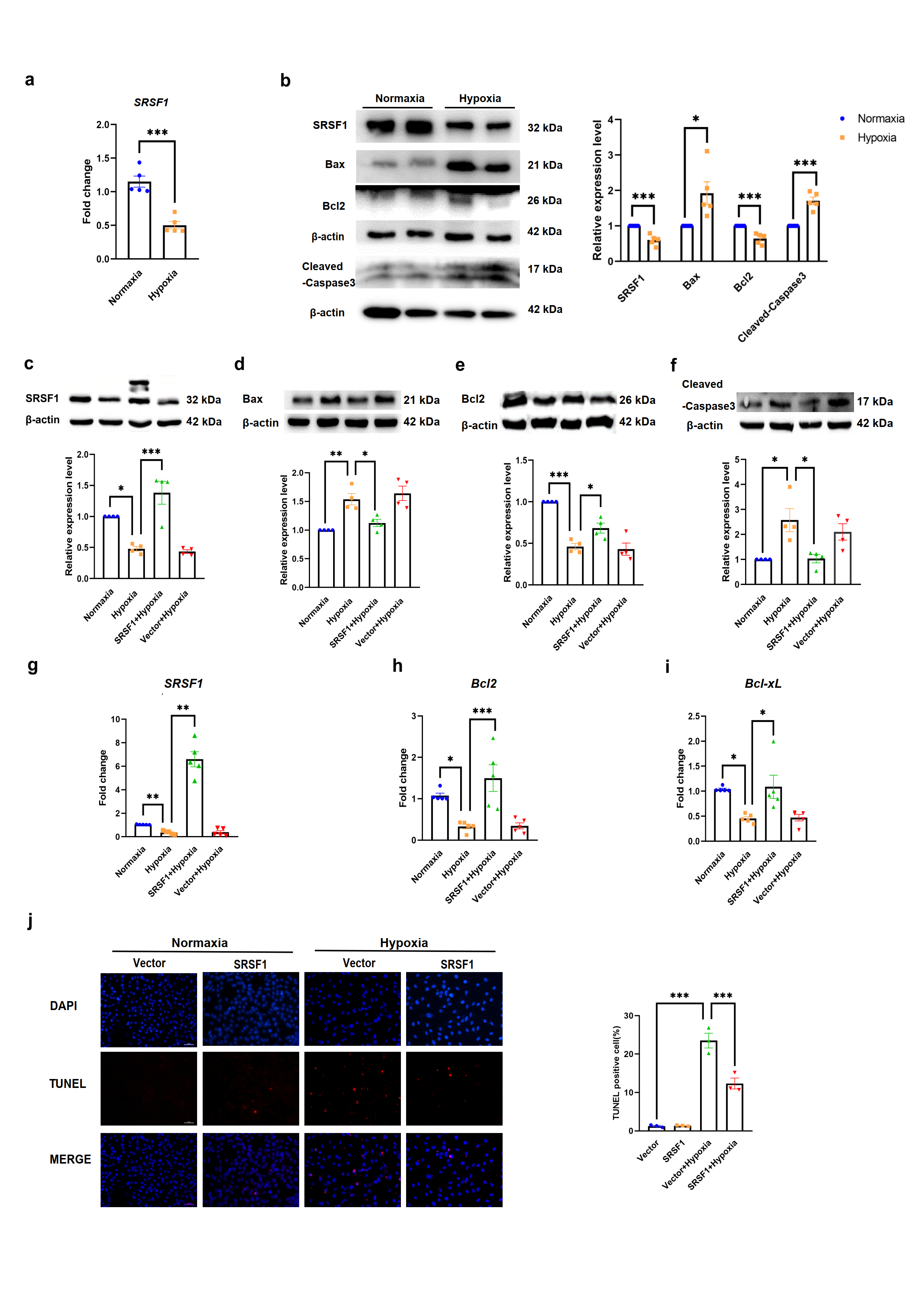
**

**Figure S2. Over-expression of SRSF1 attenuates hypoxia-induced apoptosis in H9C2 cells.**

1. The mRNA expression of SRSF1 in H9C2 cells treated with hypoxia for 24 hours (n=5). **b.** Western blot analyses of SRSF1, Bax, Bcl2, and Cleaved-Caspase3 protein levels in normaxia and hypoxia-treated (n=5). **c-f.** Forced expression of SRSF1 reversed the hypoxia-induced increase in Bax, Cleaved-Caspase3 protein expression and the decrease in Bcl2 protein expression (n=4). **g-i.** The mRNA expression of SRSF1, Bcl2, and Bcl-xL in SRSF1 overexpression cells under hypoxia treatment (n=5). **j.** TUNEL staining assay in SRSF1 overexpression cells under hypoxia treatment (scale bar = 50 μm, n=3). β-actin was used as an internal control. Data are expressed as mean ± SEM; **P* < 0.05; ***P* < 0.01; ****P* < 0.001.

**
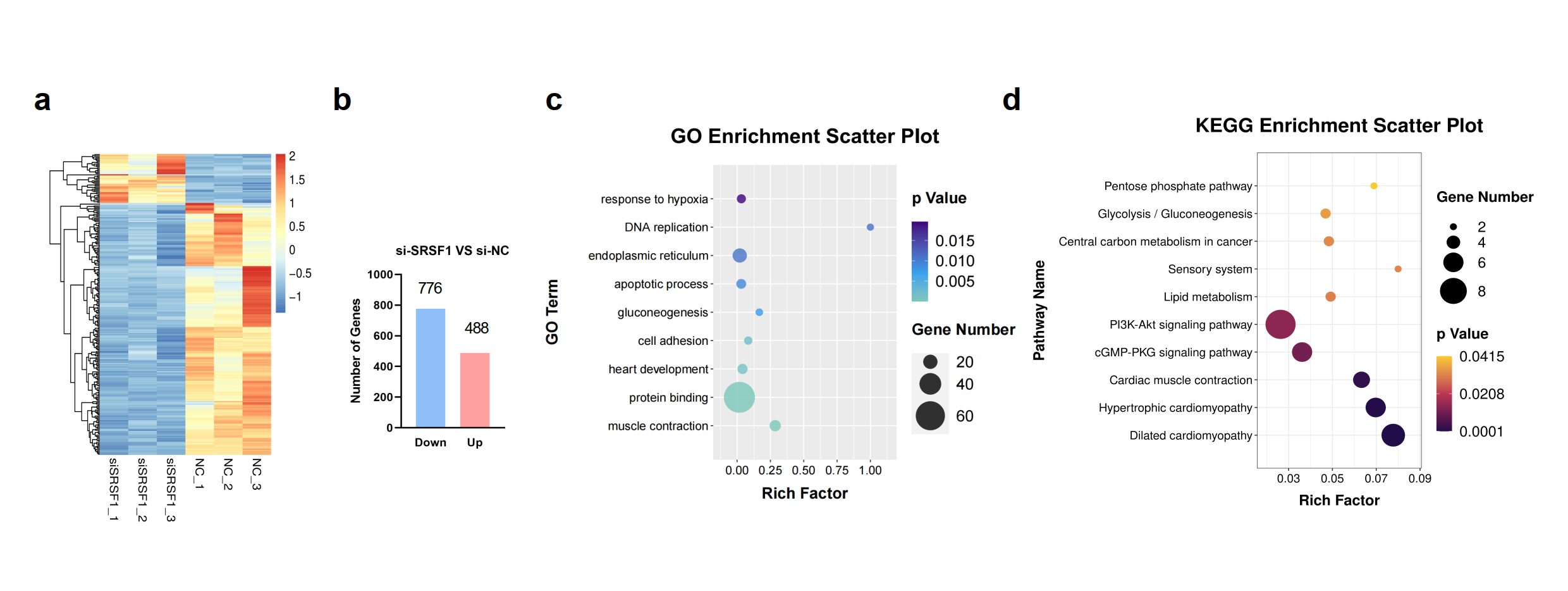
**

**Figure S3. Differences in gene expression mediated by SRSF1 knockout.**

**a-b.** Heatmap showing mRNA levels of the differentially expressed genes in SRSF1 knockdown cells, up-regulated genes (488) are shown in red, and down-regulated genes (776) are shown in blue. **c-d.** GO and KEGG enrichment analyses of differentially expressed genes in SRSF1 knockdown cells.

**
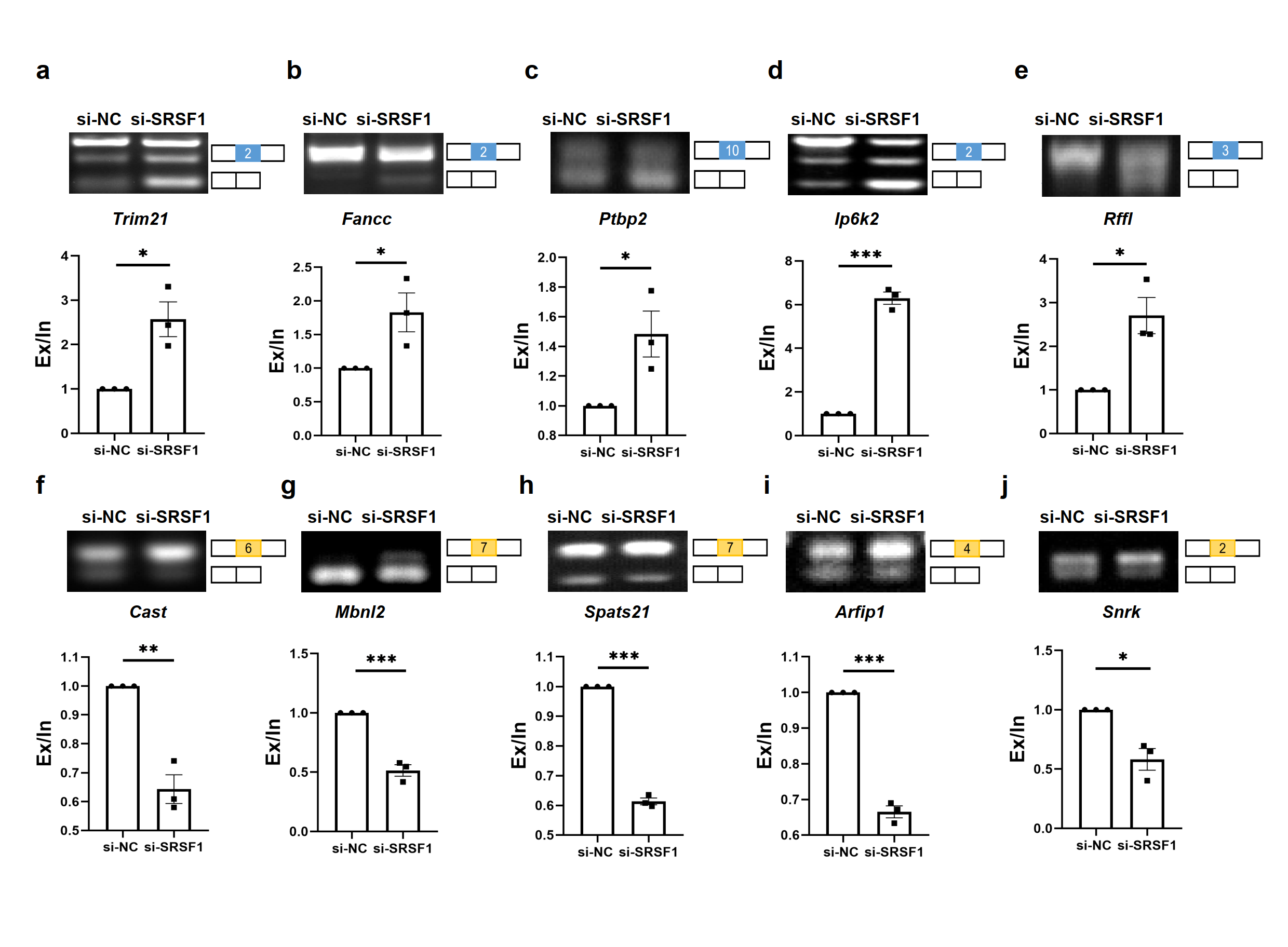
**

**Figure S4. Splicing events mediated by SRSF1 knockout.**

**a-j.** Additional validation of genes with AS changes regulated by SRSF1 knockdown. The quantification of RNA products is expressed as exclusion/inclusion (Ex/In). SRSF1 knockout mediated exon exclusion is marked in blue, whereas inclusion is in yellow (n=3). Data are expressed as mean ± SEM; **P* < 0.05; ***P* < 0.01; ****P* < 0.001.

**
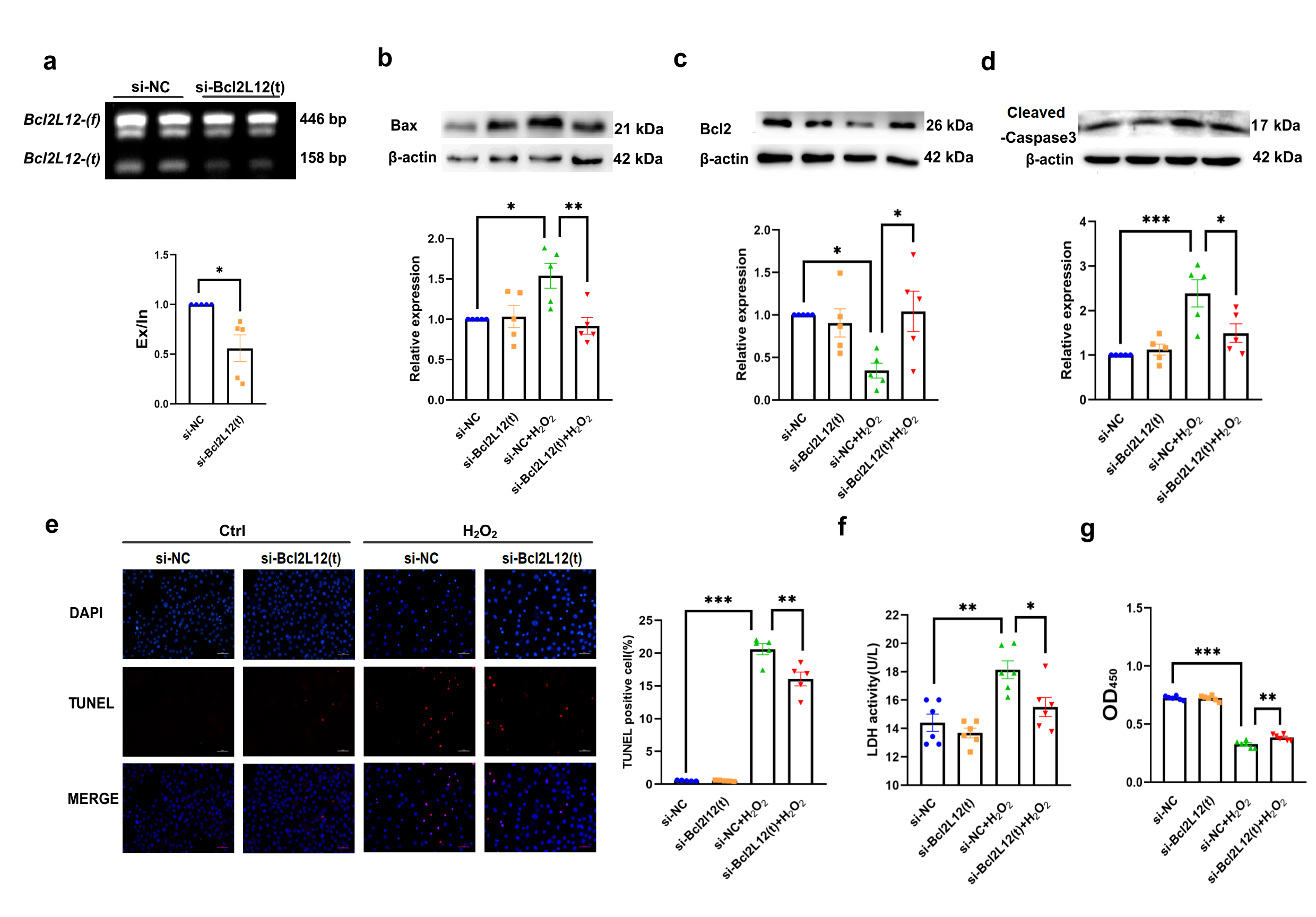
**

**Figure S5. The effect of Bcl2L12(t) knockdown in H9C2 cells treated with H_2_O_2_.**

**a.**Validation of knockout efficiency of Bcl2L12(t) mRNA level is shown using RT-PCR and gel electrophoresis (n=5). **b-d.** The protein levels of Bax, Bcl2, and Cleaved-Caspase3 in H9C2 cells transfected with si-Bcl2L12(t) after H_2_O_2_ treatment (n=5). **e.** TUNEL staining of Bcl2L12(t) knockdown cells after H_2_O_2_ treatment is displayed (scale bar = 50 μm, n=5). **f-g.** The LDH level and the cell viability of Bcl2L12(t) knockdown cells after H_2_O_2_ treatment are shown (n=6). β-actin was used as an internal control. Data are expressed as mean ± SEM; **P* < 0.05; ***P* < 0.01; ****P* < 0.001.

**
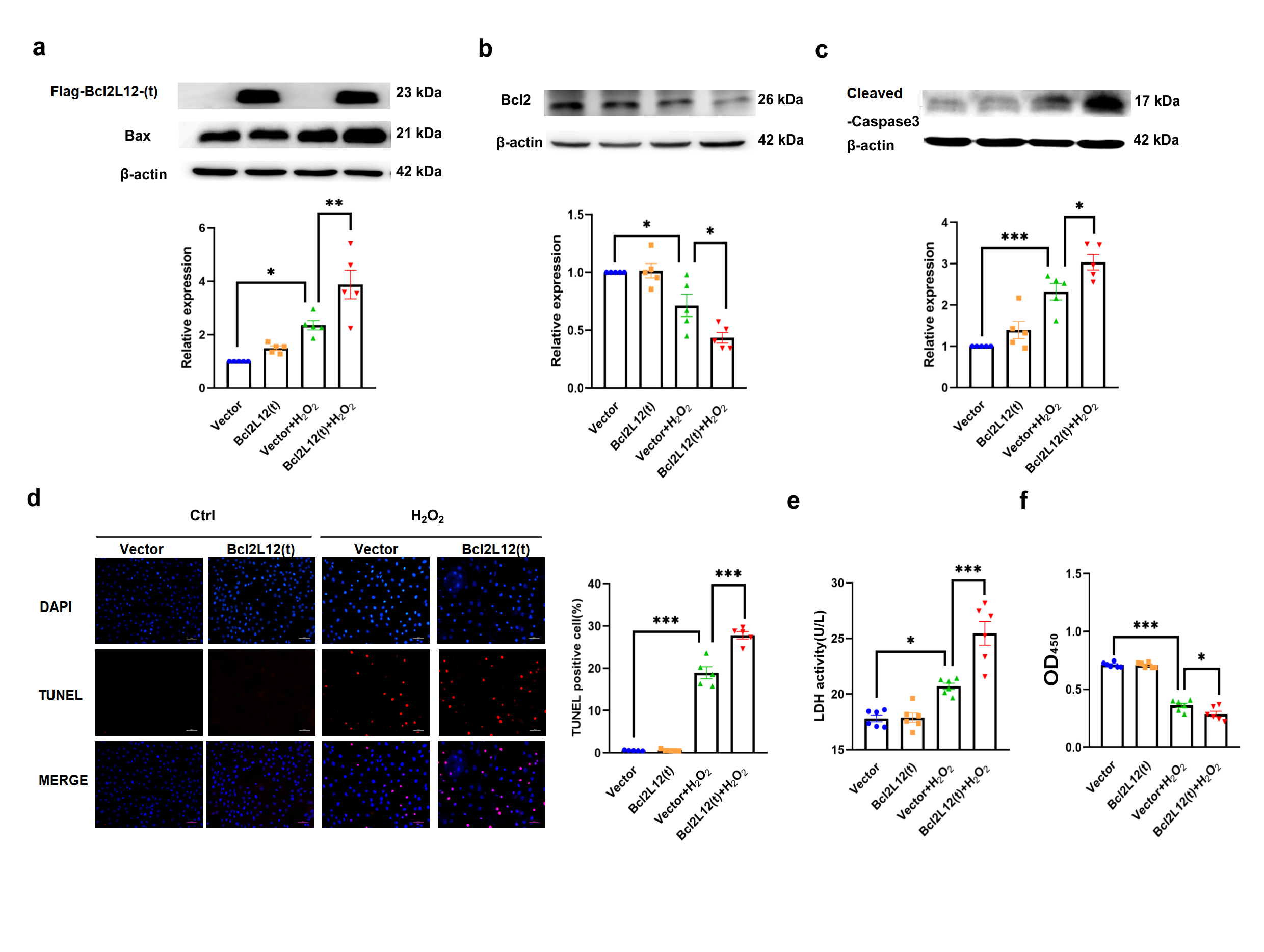
**

**Figure S6. The effect of Bcl2L12(t) overexpression in H9C2 cells treated with H_2_O_2_.**

**a-c.** The protein levels of Bax, Bcl2 and Cleaved-Caspase3 in H9C2 cells transfected with Bcl2L12(t) overexpression plasmids after H_2_O_2_ treatment. The successful expression of Bcl2L12(t) was verified by the over-expression of Flag (n=5). **d.** TUNEL staining of Bcl2L12(t) overexpression cells after H_2_O_2_ treatment is displayed (scale bar = 50 μm, n=5). **e-f.** The LDH level and cell viability of Bcl2L12(t) overexpression cells after H_2_O_2_ treatment are presented (n=6). β-actin was used as an internal control. Data are expressed as mean ± SEM; **P* < 0.05; ***P* < 0.01; ****P* < 0.001.

**
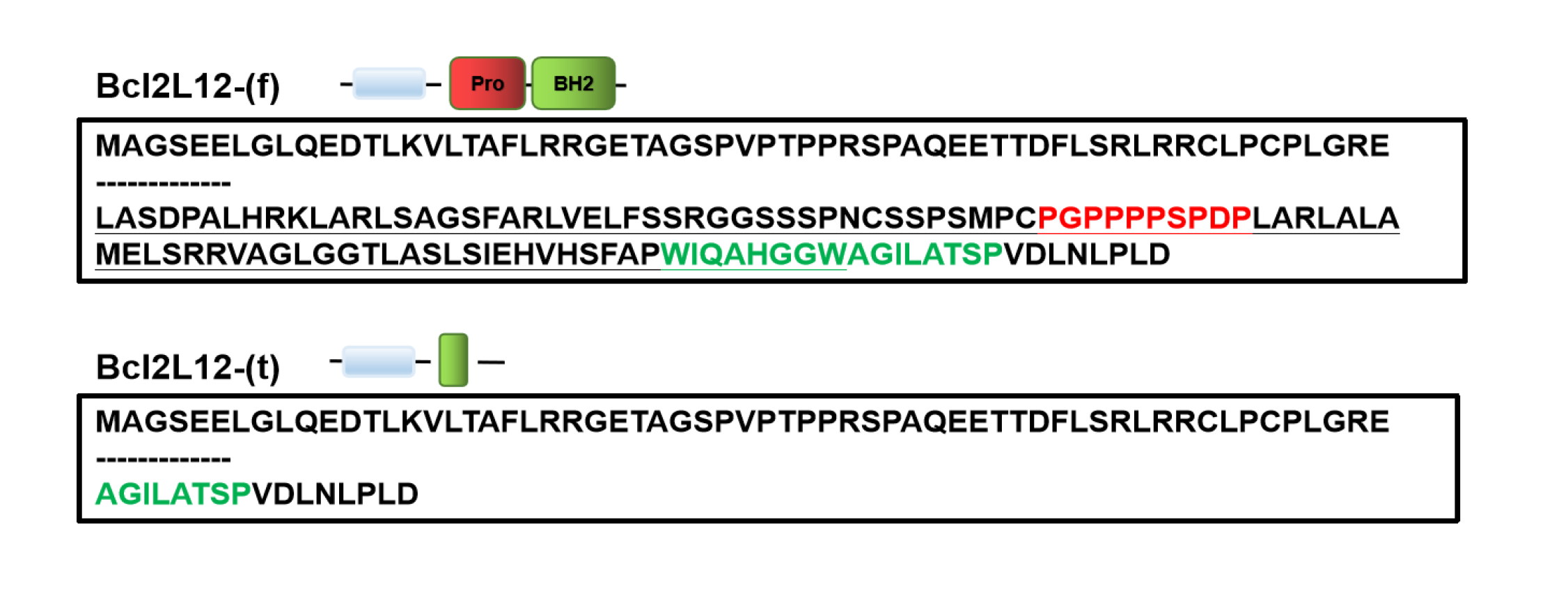
**

**Figure S7. Schematic of the protein structures of** **Bcl2L12(f) and Bcl2L12(t).**

The legend presents the amino acid sequence and structure diagram of the protein encoded by the mRNA of Bcl2L12(f) and Bcl2L12(t) subtypes. The red color represents the PPXPXP proline ring, and the green color represents the BH domain.

**
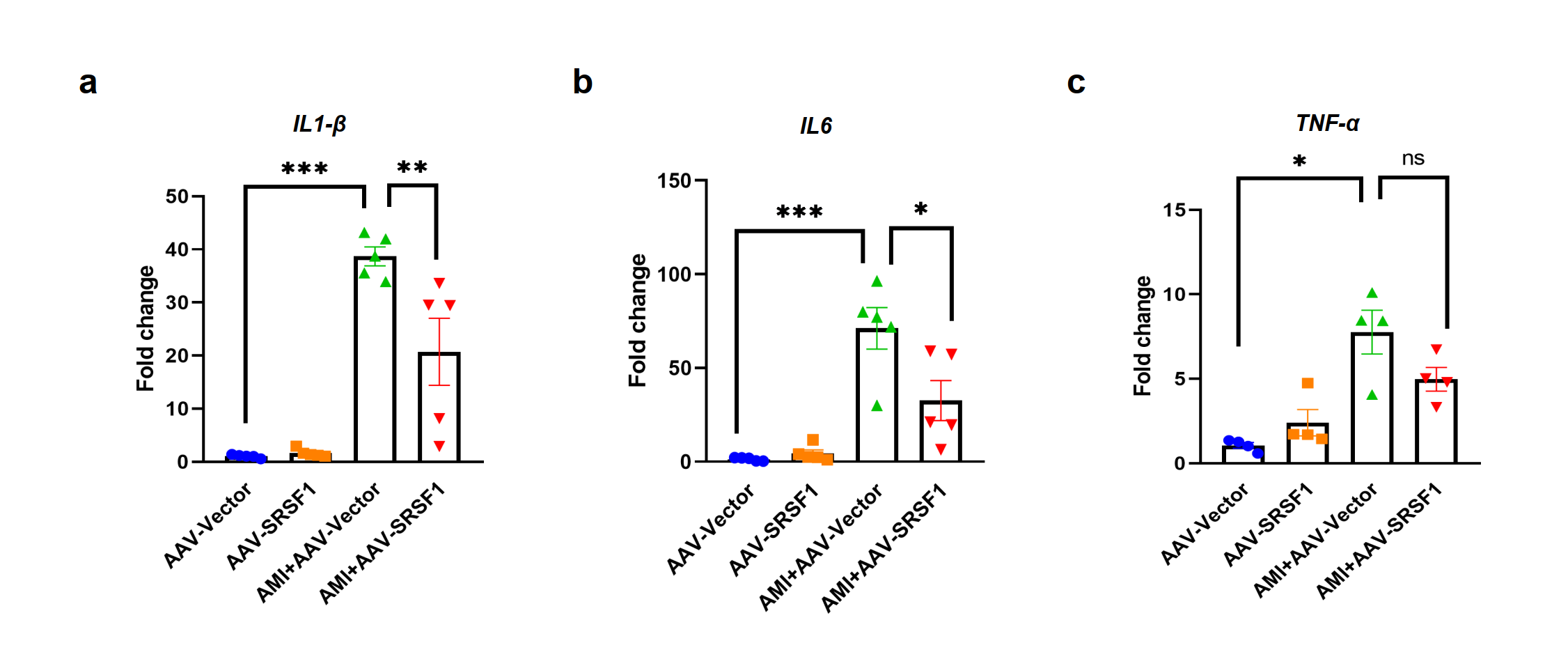
**

**Figure S8. The effect of SRSF1 on inflammatory factors in the heart of AMI mice.**

**a-c.** Expression of IL-1β, IL-6 and TNF-α at mRNA level in heart tissues of SRSF1 overexpressed AMI mice (n=4-5). Data are expressed as mean ± SEM; **P* < 0.05; ***P* < 0.01; ****P* < 0.001.

**
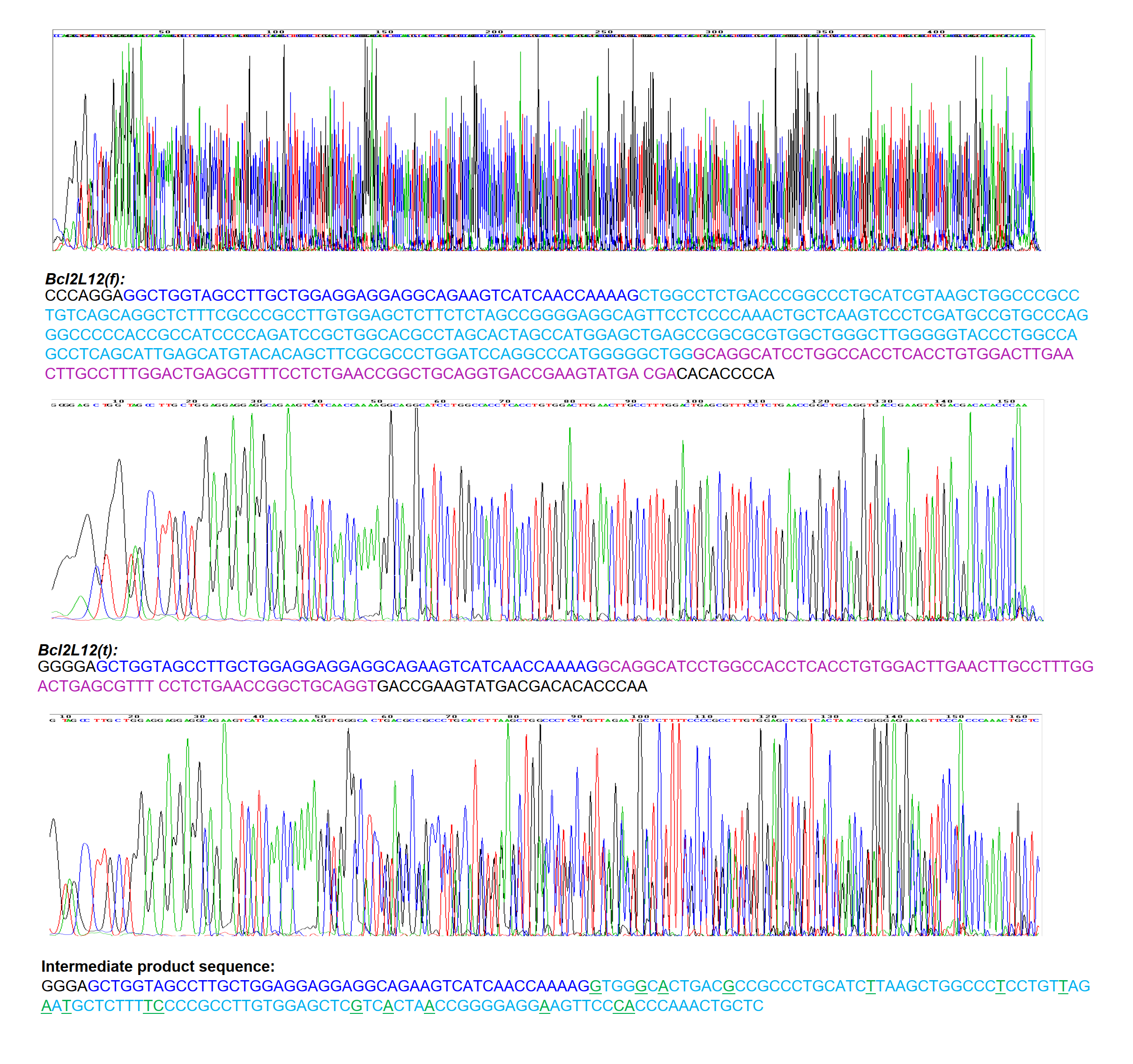
**

**Figure S9. Schematic of the mRNA structures of Bcl2L12(f) and Bcl2L12(t).**

The sequencing demonstrats the mRNA products of *Bcl2L12(f)*, *Bcl2L12(t)* and the middle bind. Blue color represents exon6, cyan color represents exon7, purple color represents exon8, and green color represents the mutated A-set peak.
