# Supplementary material for: Splicing factor SRSF1 attenuates cardiomyocytes apoptosis via regulating alternative splicing of Bcl2L12
Source: Cell Biosci. 2024 Nov 22;14:142. doi: 10.1186/s13578-024-01324-3 (PMC11585136; doi:10.1186/s13578-024-01324-3)
Supplement: Supplementary file 2 — Supplementary Material 2 [file 13578_2024_1324_MOESM2_ESM.docx]

**Supplementary table**

**Supplemental Table 1. Sequences of siRNAs**

| **Name** | **Sequences** |
| --- | --- |
| si-SRSF1-351-sense | 5’-GCUACGAUUACGACGGCUATT-3’ |
| si-SRSF1-351-antisense | 5’-UAGCCGUCGUAAUCGUAGCTT-3’ |
| si-SRSF1-174-sense | 5’-GCAUCUACGUGGGUAACCUTT-3’ |
| si-SRSF1-174-antisense | 5’-AGGUUACCCACGUAGAUGCTT-3’ |
| si-SRSF1-547-sense | 5’-GCGUGAAGCAGGUGAUGUATT-3’ |
| si-SRSF1-547-antisense | 5’-UACAUCACCUGCUUCACGCTT-3’ |
| si-Bcl2l12-(t)-sense | 5’-GUCAUCAACCAAAAGGCAGTT-3’ |
| si-Bcl2l12-(t)-antisense | 5’-CUGCCUUUUGGUUGAUGACTT-3’ |
| si-NC-sense | 5’-UUCUCCGAACGUGUCACGUTT-3’ |
| si-NC-antisense | 5’-ACGUGACACGUUCGGAGAATT-3’ |

**Supplemental Table 2. Sequences of qRT-PCR primers**

| **Name** | **Sequences** |
| --- | --- |
| *SRSF1*-Forward | 5’-AACAACGACTGCCGCATCTAC-3’ |
| *SRSF1*-Rerverse | 5’-TCGATGTCCTTGGTTCGGATA-3’ |
| *Bcl2*-Forward | 5’-GCTACCGTCGTGACTTCGC-3’ |
| *Bcl2*-Rerverse | 5’-CCCCACCGAACTCAAAGAAGG-3’ |
| *GAPDH*-Forward | 5’-CCATCTTCCAGGAGCGAGAT-3’ |
| *GAPDH*-Rerverse | 5’-AAGACGCCAGTAGACTCCAC-3’ |
| *β-actin*-Forward | 5’-CCACCATGTACCCAGGCATT-3’ |
| *β-actin*-Rerverse | 5’-CGGACTCATCGTACTCCTGC-3’ |
| *Bcl-xL*-Forward | 5’-ACATCCCAGCTTCACATAACCC-3’ |
| *Bcl-xL*-Rerverse | 5’-TCCCGTAGAGATCCACAAAAGT-3’ |
| *1L-1β*-Forward | 5’-ATGGCAACTGTTCCTGAACTCAACT-3’ |
| *1L-1β*-Rerverse | 5’-CAGGACAGGTATAGATTCTTTCCTTT-3’ |
| *1L-6*-Forward | 5’-AGGATACCACTCCCAACAGACCT-3’ |
| *1L-6*-Rerverse | 5’-CAAGTGCATCATCGTTGTTCATAC-3’ |
| *TNF-α*-Forward | 5’-ATACACTGGCCCGAGGCAAC-3’ |
| *TNF-α*-Rerverse | 5’-CCACATCTCGGATCATGCTTTC-3’ |

**Supplemental Table 3. Sequences of RT-PCR primers**

| **Name** | **Sequences** |
| --- | --- |
| *Bcl2l12*-1-Forward | 5’-GAGGAGGCAGAAGTCATCAACC-3’ |
| *Bcl2l12*-1-Rerverse | 5’-CCACCTCCATGCACTTACTC-3’ |
| *Bcl2l12*-2-Forward | 5’-AGGCTGGTAGCCTTGCTGGA-3’ |
| *Bcl2l12*-2-Rerverse | 5’-GCCCTGAAGAATGTGTGTCGTCAT-3’ |
| *Bcl2l12*-exon7-Forward | 5’-CATCGTAAGCTGGCCCG-3’ |
| *Bcl2l12*-exon-Rerverse | 5’-CGAAGCTGTGTACATGCTC-3’ |
| Minigene-*Bcl2l12*-Forward | 5’-GGAGAAGGAAGCCCTGCTAA-3’ |
| Minigene-*Bcl2l12*-Rerverse | 5’-AGCCAGAAGTCAGATGCTCAA-3’ |
| *Trim21*-Forward | 5’-CCATTAGACTGCGGGCTTGC-3’ |
| *Trim21*-Rerverse | 5’-GCTTCTCGGCCAACTCTTTCC-3’ |
| *Mtmr3*-Forward | 5’-CAGCTCTCTGCGCTTCAATGG-3’ |
| *Mtmr3*-Rerverse | 5’-CTTGCACACTCGACTGGGTTC-3’ |
| *Fancc*-Forward | 5’-CAAGTTCCGTGAGGTCGTGC-3’ |
| *Fancc*-Rerverse | 5’-CCGTTCCAGGATTGCATCAGAATC-3’ |
| *Nfib*-Forward | 5’-AGAGACCCATCCTTCCTGCAT-3’ |
| *Nfib*-Rerverse | 5’-TAGTGACCGGAAGTGCTGC-3’ |
| *Mbnl2*-Forward | 5’-TTCACCCTCCTGCACACTTGC-3’ |
| *Mbnl2*-Rerverse | 5’-TGCTGGTAGTGCAAGACGC-3’ |
| *Myef2*-Forward | 5’-ATTGAGTCGAGGCTTTGGCG-3’ |
| *Myef2*-Rerverse | 5’-CTCCTTTCCAGTATGGCTCCGA-3’ |
| *Ip6k2*-Forward | 5’-CGCAGCCATTTTGGTGGAAGAGA-3’ |
| *Ip6k2*-Rerverse | 5’-GCACCCAGTCCTTGGGACTCTT-3’ |
| *Med7*-Forward | 5’-CTACGGGAAAAGCTAGAGCT-3’ |
| *Med7*-Rerverse | 5’-CTACGGGAAAAGCTAGAGCT-3’ |
| *Pkd1*-Forward | 5’-GCCTTGTGTCCAGTGTTGTAGT-3’ |
| *Pkd1*-Rerverse | 5’-GGTCATTCTTCACTTGCCCAG-3’ |
| *Ubn1*-Forward | 5’-ATTGTCACAGGTCCAGCCC-3’ |
| *Ubn1*-Rerverse | 5’-CATGTTGGGCTTCCTCCTAGA-3’ |
| *Snrk*-Forward | 5’-ATGATTAGCCCTGGGTGCG-3’ |
| *Snrk*-Rerverse | 5’-GCTTGAACCCTGCCATGCTG-3’ |
| *Spats2l*-Forward | 5’-AGGAACCACCAAGGGCTCAG-3’ |
| *Spats2l*-Rerverse | 5’-AGAACTGTCCACCTCTTCCTTG-3’ |
| *Cast*-Forward | 5’-GAAGCCAAAGGAAGGGTCAGAG-3’ |
| *Cast*-Rerverse | 5’-CCAGCCACACTCTCTCCATCAG-3’ |
| *Rffl*-Forward | 5’-GACTTCTGCATGACTTGCTCC-3’ |
| *Rffl*-Rerverse | 5’-GAGTTGCGAGCCAGGATCTC-3’ |
| *Arfip1*-Forward | 5’-ACTGAAGCAGGAGCATCCCA-3’ |
| *Arfip1*-Rerverse | 5’-GGTCCACCTTTTGTGTGTGTTC-3’ |
| *Ptbp2*-Forward | 5’-GGGCTCTCAGTCCTTTGGCT-3’ |
| *Ptbp2*-Rerverse | 5’-GGCATCGGCCATTTGTATCAG-3’ |
| *Trpc1*-Forward | 5’-CTGAAGGATGTGCGAGAGGTG-3’ |
| *Trpc1*-Rerverse | 5’-CCATCGTTGTTGAGTATTCCGG-3’ |
| *Pkd1*-Forward | 5’-GCCTTGTGTCCAGTGTTGTAGT-3’ |
| *Pkd1*-Rerverse | 5’-GGTCATTCTTCACTTGCCCAG-3’ |
| *Rhebl1*-Forward | 5’-GTTCCTTGCGTTTGCCGG-3’ |
| *Rhebl1*-Rerverse | 5’-CAAGCACGTAACCATGGACC-3’ |

**Supplementary table 4. Alternative splicing events information regulated by SRSF1.**

| **gene** | **chr** | **exonStart** | **exonEnd** | ***P* Value** | **IncLevel Difference** |
| --- | --- | --- | --- | --- | --- |
| *"Mtmr3"* | chr14 | 84706454 | 84706565 | 2.49E-14 | -0.313 |
| *"Trim21"* | chr1 | 167699688 | 167700120 | 4.01E-09 | -0.444 |
| *"Bcl2l12"* | chr1 | 100986699 | 100986987 | 3.21E-03 | -0.143 |
| *"Fancc"* | chr17 | 920101 | 920298 | 3.68E-05 | -0.34 |
| *"Nfib"* | chr5 | 100454026 | 100454115 | 3.68E-02 | -0.396 |
| *"Ptbp2"* | chr2 | 223267532 | 223267566 | 1.57E-02 | -0.441 |
| *"Trpc1"* | chr8 | 103547986 | 103548141 | 1.86E-02 | -0.381 |
| *"Myef2"* | chr3 | 117362732 | 117362804 | 6.19E-04 | -0.243 |
| *"Ip6k2"* | chr8 | 117589226 | 117589600 | 3.33E-05 | -0.233 |
| *"Rffl"* | chr10 | 70164811 | 70164895 | 2.23E-08 | -0.429 |
| *"Cast"* | chr2 | 1508220 | 1508334 | 4.55E-08 | 0.307 |
| *"Sp110"* | chr9 | 92605962 | 92606040 | 1.45E-05 | 0.485 |
| *"Rhebl1"* | chr7 | 140555751 | 140555823 | 1.44E-03 | 0.445 |
| *"Mbnl2"* | chr15 | 105763421 | 105763475 | 6.03303E-06 | 0.49 |
| *"Pkd1"* | chr10 | 13953436 | 13953550 | 2.06E-03 | 0.191 |
| *"Ubn1"* | chr10 | 10694529 | 10694619 | 1.54E-05 | 0.22 |
| *"Spats2l"* | chr9 | 64798317 | 64798524 | 9.12E-07 | 0.239 |
| *"Arfip1"* | chr2 | 183535765 | 183535861 | 2.82E-08 | 0.342 |
| *"Med7"* | chr10 | 31534893 | 31535062 | 1.55E-03 | 0.305 |
| *"Snrk"* | chr8 | 130762664 | 130762715 | 2.58E-03 | 0.249 |
